# Development, scrutiny, and modulation of transient reporter gene assays of the xenobiotic metabolism pathway in zebrafish hepatocytes

**DOI:** 10.1007/s10565-021-09659-0

**Published:** 2021-10-15

**Authors:** Sebastian Lungu-Mitea, Yuxin Han, Johan Lundqvist

**Affiliations:** Department of Biomedicine and Veterinary Public Health, Swedish University of Agricultural Sciences, Box 7028, 750 07 Uppsala, Sweden

**Keywords:** Bioassays, Reporter gene assays, Transient transfection, Fish cell lines, Squelching

## Abstract

The “toxicology in the twenty-first century” paradigm shift demands the development of alternative in vitro test systems. Especially in the field of ecotoxicology, coverage of aquatic species-specific assays is relatively scarce. Transient reporter gene assays could be a quick, economical, and reliable bridging technology. However, the user should be aware of potential pitfalls that are influenced by reporter vector geometry. Here, we report the development of an AhR-responsive transient reporter-gene assay in the permanent zebrafish hepatocytes cell line (ZFL). Additionally, we disclose how viral, constitutive promoters within reporter-gene assay cassettes induce squelching of the primary signal. To counter this, we designed a novel normalization vector, bearing an endogenous zebrafish-derived genomic promoter (zfEF1aPro), which rescues the squelching-delimited system, thus, giving new insights into the modulation of transient reporter systems under xenobiotic stress. Finally, we uncovered how the ubiquitously used ligand BNF promiscuously activates multiple toxicity pathways of the xenobiotic metabolism and cellular stress response in an orchestral manner, presumably leading to a concentration-related inhibition of the AhR/ARNT/XRE-toxicity pathway and non-monotonous concentration–response curves. We named such a multi-level inhibitory mechanism that might mask effects as “maisonette squelching*.*”

**Graphical abstract:**

A transient reporter gene assay in zebrafish cell lines utilizing endogenous regulatory gene elements shows increased in vitro toxicity testing performance.

Synthetic and constitutive promotors interfere with signal transduction (“squelching”) and might increase cellular stress (cytotoxicity).

The squelching phenomenon might occur on multiple levels (toxicity pathway crosstalk and normalization vector), leading to a complete silencing of the reporter signal.

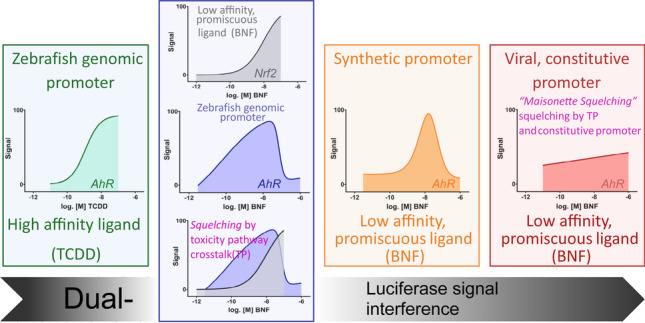

**Supplementary Information:**

The online version contains supplementary material available at 10.1007/s10565-021-09659-0.

## Introduction

Within the last two decades, several chemical regulation bills (The European Parliament and the Council 2003, 2006, 2009; US EPA 2016) were conceptualized, ratified, and utilized on an international level, intending the proper protection of humankind and the surrounding environment from anthropogenically derived compounds. Unfortunately, increasing demands in toxicological in vivo testing triggered the dilemma of weighing risk assessment against economic feasibility and ethical standards (Goldberg 2010; Hartung 2010, 2011). This development contradicts efforts made within the scientific community to promote the acceptance and use of the “3Rs” (Russell and Burch 1959; Lillicrap et al. 2016). The use of alternative or new approach methods (NAMs), such as in vitro and in silico based applications, for the detection and measurement of “toxicity pathways” (TPs), has been postulated as a primary concept of the “toxicology in the twenty-first century” (Tox21) framework (NRC 2007; Collins et al. 2008; Whelan and Andersen 2013; Kleensang 2014). A TP is defined as a sequence of intracellular events, which maintain cellular homeostasis under physiological conditions, but once perturbed by a xenobiotic, may lead to adverse effects on the cellular and, beyond, on the organismal level of biological complexity (Whelan and Andersen 2013). Shortly after, TPs were augmented by the adverse outcome pathway (AOP) concept (Ankley et al. 2010), which projects the TP concept onto the population and community scale and adds elements of network plasticity.

TPs of the xenobiotic metabolism are among the best studied. Ligand-dependent recruitment of the aryl hydrocarbon receptor (AhR) transcription factor by xenobiotics, the translocation sequestered by its dimerization partner the aryl hydrocarbon receptor nuclear translocator (ARNT), binding to the xenobiotic response element (XRE), and synthesis of downstream phase I and II xenobiotic metabolism enzymes have extensively been researched and are well-documented in the literature (reviewed in Schmidt and Bradfield 1996)). The AhR is known to be promiscuous. Firstly, in the sense that it binds exogenous and endogenous ligands with different affinities (Soshilov and Denison 2014; Tagliabue et al. 2019), and secondly, in terms of downstream activation and crosstalk with non-canonical pathways by dimerization with alternate translocators and transcriptional co-factors (reviewed in Denison and Faber 2017)).

Reporter gene assays are an ubiquitous tool for screening TPs (Zacharewski 1997; Ankley et al. 1998; Mueller 2004; Leusch and Snyder 2015), given their intrinsic ability to define molecular initiating events (MIE) and mechanism of action (MOA). Reporter gene assays comprise pro and eukaryotic cellular systems bearing stably or transiently introduced reporter gene cassettes. The cassettes are composed of a specific genomic or synthetic TP-related response element, fused to a reporter enzyme coding sequence, such as luciferase or the green fluorescence protein. Upon activation of the TP-specific response element, the utilized reporter is synthesized in parallel to the TP-specific target genes and enzymes. The reporter signal is quantifiable, thus disclosing the turnover of the TP-specific target genes to the investigator. Some reporter gene and catalytic enzyme assays for assessing xenobiotic metabolism-related toxicity have been established, mainly in rodent cell lines, and proofed successful in various fields of toxicity testing (reviewed in Eichbaum et al. 2014)).

However, within ecotoxicology, coverage of TPs by species-specific reporter assays is relatively scarce, and a couple of strategy papers encouraged their development within the last years (EURL-ECVAM 2014; Halder et al. 2014; Worth et al. 2014). Nonetheless, the AhR/ARNT/XRE TP is the best established in terms of available assays, especially in comparison to other TPs. A few reporter gene assays have been developed in rainbow trout (*Oncorhynchus mykiss*) (Richter et al. 1997; Villeneuve et al. 1999) and zebrafish (*Danio rerio*) cell lines (Carvan et al. 2000; Mattingly et al. 2001; Yang et al. 2016; Chen and Chan 2017; Zhou et al. 2017). The zebrafish offers a toxicity testing platform of great potential. The fish embryo test (FET) was one of the first in vitro assays to gain partly regulatory acceptance (OECD 2013; Belanger et al. 2013). Further, the zebrafish is a standard test species in developmental molecular biology, and a plethora of biotechnological tools is available. Thus, a few transgenic zebrafish strains have already been established and can be used in parallel to cellular assays to identify and measure TPs, also facilitating in vitro to in vivo extrapolation (IVIVE) studies (reviewed in Garcia et al. 2016)). Beyond, all AhR/ARNT/XRE pathway components were successfully characterized in zebrafish (Tanguay et al. 1999, 2000; Andreasen et al. 2002; Zeruth and Pollenz 2005, 2007; Hahn et al. 2017), hence, enabling the investigator to draw mechanistic conclusions. The demand for reliable reporter gene assays indicating critical toxicity endpoints within ecotoxicology is on the rise, given the fact that the current European Water Framework Directive (WFD) is approaching its deadline in 2027. A potential continuation of the directive will propose the addition of effect-based and directed tools (bioassays) to accompany classical chemical analysis in environmental screenings (Wernersson et al. 2015; Brack et al. 2017, 2018, 2019). The discussion has yet been indecisive regarding to what amount established mammalian assays should be incorporated or if assays derived from aquatic organisms are more representative (Lillicrap et al. 2016; Neale et al. 2020).

In comparison to stably transfected constructs, transient reporter gene assays have the advantage of being more flexible and more comfortable to handle regarding logistics and maintenance. One primary permanent cell line can be used and transiently transfected with different constructs, thus covering the assessment of various TPs. Further, the distribution of reporter-containing plasmid DNA is more straightforward than the distribution of living cells. Finally, reporter constructs on plasmid DNA are easily accessible and can be modified by the investigator to fit their specific needs. However, there might be a misconception regarding reporter gene assays’ overall reliability and correct application. Notably, stable or transient transgenesis of non-endogenous genetic constructs will interfere with the host organism on genomic, epigenetic, and phenotypic levels. Thus, the manipulated model system might be impacted beyond the inquired pathway’s response and produce nonspecific results (reviewed in Stepanenko and Heng 2017). Recently, we assessed the issue of transient transgenesis in zebrafish cell lines, displaying significant differences in reporter gene assay potency, efficacy, and reliability in regard to plasmid vector geometry (Lungu-Mitea and Lundqvist 2020). Transient transfection is impacted synergistically in terms of vector sizes, vector backbones, gene-regulatory units, tissue origin, transfection method, and dual luciferase signal normalization. Further, we proposed strategies to account for spurious TP regulation and how to approach the design of transient TP-related reporter gene assays.

This study aimed to design a potent transient reporter gene assay of the AhR-related xenobiotic metabolism TP in the permanent zebrafish hepatocyte cell line (ZFL) (Ghosh et al. 1994). In the perspective of our previous work, we wanted to examine spurious up or downregulation of the TP-mediated signal in transiently transfected cells under stress by chemical exposure. Thus, we hypothesized that the potency and efficacy of a compound, as recorded by the reporter gene assay, might be affected by the vector geometry, most likely by the inherent gene-regulatory units. Additionally, we hypothesized to encounter synergistically acting cellular stress due to synchrony of transfection and exposure, which might alternate the recoded signal in a specific way.

## Materials and methods

### Chemicals

Known AhR-agonists β-naphthoflavone (BNF; ≥ 98% purity; Sigma-Aldrich, Steinheim, Germany) and 2,3,7,8-tetrachlorodibenzo-p-dioxin (TCDD; 10 μg/mL in toluene; Supelco analytical standard; Sigma-Aldrich, Steinheim, Germany) were used in all experiments. BNF powder was dissolved in dimethyl sulfoxide (DMSO; anhydrous, ≥ 99.9% purity, sterile; Sigma-Aldrich, Steinheim, Germany) to a 20-mM stock concentration. The toluene solvent in the TCDD vial was evaporated, and crystalized TCDD redissolved in DMSO to a 32-µM stock concentration. Both BNF and TCDD stock solutions were aliquoted into minor volumes to thaw only one vial per week/experiment. Stocks were stored at – 20 °C in a desiccator.

### Cell culturing

The zebrafish liver cell line (CVCL_3276) (Ghosh et al. 1994) was purchased from ATCC (Mannassas, USA). A nutrition medium consisting of 50% (v/v) Leibovitz’s L-15 (Sigma-Aldrich, Steinheim, Germany), 35% (v/v) Dulbecco’s modified Eagle’s medium (Gibco, Paisley, UK), 15% (v/v) Ham’s Nutrient Mixture F-12 (Gibco, Paisley, UK), and phenol red, supplemented by 150 mg/L sodium bicarbonate (Gibco, Paisley, UK), 15 mM HEPES (Gibco, Paisley, UK), 10 μg/mL bovine insulin (Sigma-Aldrich, Steinheim, Germany), 50 ng/mL mouse EGF (Sigma-Aldrich, Steinheim, Germany), and 5% (v/v) fetal bovine serum (Gibco, Paisley, UK), was used for cultivation. The cells were cultured in a humidified environment at 28 °C and atmospheric CO_2_. Further, cells were passaged weekly in a 1:20 subcultivation ratio. Phosphate buffered saline (PBS, pH 7.4; Medicago, Uppsala, Sweden) was used for washing and 0.25% (w/v) trypsin–EDTA (Sigma-Aldrich, Steinheim, Germany) for cell detachment. All experiments were conducted within passage numbers 5 to 32.

### Plasmids and cloning

The **pGL4.43[luc2p/XRE/hygro]** plasmid was acquired from Promega (Madison, USA). The latter consists of a pGL4 backbone including an ampicillin resistance gene, a gene for hygromycin resistance, and three copies of a synthetic xenobiotic response element (XRE) driving transcription of the Firefly luciferase reporter gene luc2P (*Photinus pyralis*). The **pGudluc7.5** plasmid was donated by Michael S. Denison (University of California, Davies, USA). The latter consists of a **pGL3** backbone, 20 XRE copies from the genomic promoter region of the *mCyp1A1* gene upstream of an MMTV viral promoter, and the Firefly luciferase reporter gene (Denison et al. 1989; El-Fouly et al. 1994; Garrison et al. 1996; Han et al. 2004; He et al. 2011). Firefly luciferase was used as the primary reporter in this study.

Plasmids of the **pRL** and **pGL4** series were also acquired from Promega. All plasmids consist of a **pRL** or **pGL4** backbone including the cDNA encoding for Renilla luciferase reporter gene (Rluc) (*Renilla reniformis*) and a specific constitutive promoter sequence of viral origin (Table 1, Fig. S2): **pRL-null/ pGL4.70**: minimal promotor (minP); **pRL-SV40/ pGL4.73**: simian virus 40 promotor; **pRL-TK/ pGL4.74**: herpes simplex virus thymidine kinase promoter; **pRL-CMV/ pGL4.75**: cytomegalovirus promotor. Renilla luciferases were used as control/normalization signals in the following dual reporter gene assays (DLR).

**Table 1:** Used plasmid vectors, their specific promoter (+ enhancer in some cases), and the luciferase reporter. Consult Figs. S1 and S2 for the plasmid vector geometry

Plasmid vector	Promoter/enhancer	Reporter	Size [nt]
pGL4.43[luc2p/XRE/hygro]	3 × XRE (synthetic) + minP	Firefly	6067
pGudLuc7.5	20 × XRE (genomic) + MMTV	Firefly	~ 8300
pGL4.70[hRluc]	minP	Renilla	3522
pGL4.73[hRluc/SV40]	SV40	Renilla	3921
pGL4.74[hRluc/TK]	TK	Renilla	4237
pGL4.75[hRluc/CMV]	CMV	Renilla	4281
pRL-null	minP	Renilla	3320
pRL-SV40	SV40	Renilla	3705
pRL-TK	TK	Renilla	4045
pRL-CMV	CMV	Renilla	4079
pRL-null[zfEF1aPro]	zfEF1aPro + minP	Renilla	4795

The “**ZF-L Exp**.” plasmid, containing the zebrafish translation elongation factor 1 alpha promoter (zfEF1aPro), was donated by Patrick Heinrich (University of Heidelberg, Germany) (Heinrich and Braunbeck 2018). *zfEF1aPro* cDNA was initially amplified from an adult female Westaquarium strain zebrafish and cloned into the “**ZF-L Exp**” vector. Within the “**ZF-L Exp**” vector, the *zfEF1aPro* fragment (1.4 kB) is flanked by a BglII and an XhoI restriction site at the respective 5' and 3' ends, which allows easy insertion into the multiple cloning site (MCS) of the pRL-null plasmid in the forward direction. Restriction digestion, ligation, and propagation of the newly designed plasmid **pRL-null[zfEF1aPro]** (Fig. S1) are described in detail in the supplementary information (SI).

ZFL cells were also transiently co-transfected with the **pGL4.37** and **pRL-CMV** vector pair to study potential impacts on the oxidative stress response pathway after BNF exposure. A transient reporter gene assay for the assessment of oxidative stress in the ZFL cell line had been described previously (Lungu-Mitea and Lundqvist 2020), and was applied here in an identical manner. Please consult the respective chapter within the SI for more details.

### Handling, transient transfection, and exposure

ZFL cells were seeded either into white, clear-bottom 96-well microtiter plates (Corning, New York, USA; for DLR assays) or transparent 96-well microtiter plates (Corning, New York, USA; for viability assays) at a density of 1.5 × 10^5^ cells/mL, in 100 μL/well. After 24 h of incubation, cells reached a confluence of about 80%.

The transient transfection was conducted in a 2-μg transfection reagent to 1 μg plasmid DNA ratio, applying the XHP transfection reagent (Roche, Mannheim, Germany). Specific transfection optimization experiments were reported priory (Lungu-Mitea et al. 2018). The transient transfection reaction was incubated as a co-transfection in a 10:1 reporter to control ratio (0.9 μg reporter plasmid, 0.1 μg control plasmid) for 30 min, before adding the reaction mixture to the cells. Firefly luciferase-bearing vectors were used as the primary reporter plasmids, and Renilla luciferase-bearing vectors were used for normalization (control plasmid). Ten microliter per well of the transfection reaction mix was added to the cells and incubated for 24 h. Following (Lungu-Mitea and Lundqvist 2020), cells used in both DLR and viability assays were transiently transfected.

After 24 h of post-transfection incubation, cells were exposed to known AhR-agonists BNF or TCDD. Prepared aliquots of stock solutions were thawed and further titrated using the nutrition medium supplemented with 5‰ (v/v) DMSO as a solvent. Seeded cells on 96-well plates were exposed in triplicates to increasing nominal concentrations of BNF and TCDD. Different exposure regimes were applied. In terms of BNF, cells were first exposed to concentrations of 3, 30, and 300 nM. All potential co-transfection combinations were screened in this initial approach. Candidate plasmids that scored higher overall induction values were reassessed in an exposure regime of 300 pM to 1 µM BNF (0.3, 1, 3, 10, 30, 100, 300, 1000 nM). Further, cells transfected with candidate plasmid combinations were subjected to a TCDD exposure regime from 100 pM to 100 nM (0.1, 0.3, 1, 3, 10, 30, 100 nM). Cells were exposed in 100 μL/well of the specifically spiked nutrition medium. Solvent controls (negative controls) were conducted in six replicates for every specific experiment.

### Dual reporter gene assay

After 24 h of exposure to AhR-agonists, cells on white 96-well microtiter plates were lysed in 20-μL passive lysis buffer (PLB; Promega, Madison, USA), and quantitative AhR-dependent luminescence was measured via Dual-Luciferase® Reporter Assay (DLR; Promega, Madison, USA), according to the manufacturer’s protocol using an auto-injecting Infinite M1000 microplate reader (Tecan, Männedorf, Switzerland), following a flash luminescence protocol. The luciferase activity was normalized to transfection efficiency (Firefly raw light unit readout divided by Renilla raw light unit readout) and further expressed as fold change compared to the non-treated controls.

### Viability testing

In parallel to the DLR assays, MTS/BCA-multiplex assays were conducted on identically treated cells. The MTS/BCA-multiplex assay, which is described in detail in (Lungu-Mitea et al. 2021), scores NADPH-turnover and total protein amount as apical endpoints of cell viability or cytotoxicity. After 24 h exposure, the spiked nutrition medium was discharged, cells were washed once in 100 µL/well PBS, and 100 µL/well Earle’s Balanced Salt Solution (EBSS; Gibco, Paisley, UK) was added. MTS-based CellTiter 96® AQueous One Solution Cell Proliferation Assay (Promega, Madison, USA) was conducted following the manufacturer’s protocol. Seventeen percent (v/v) MTS reagent were added to the wells. After 2 h of incubation at 28 °C and atmospherical CO_2_, formazan product turnover absorbance was measured at 490 nm using an Infinite M1000 microplate reader. Afterwards, EBSS and MTS reagents were removed. Cells were washed once in 100 µL/well PBS and lysed in 20-µL PLB at RT for 30 min. Bicinchoninic Acid Protein Assay (BCA, Sigma-Aldrich, St. Louis, USA) was conducted following the manufacturer’s protocol. One hundred eighty microliters of BCA-reagent was added to every well, plates were agitated shortly, and incubated for 20 min at 60 °C. After cooling down for 15 min at RT, absorbance was measured at 562 nm using an Infinite M1000 microplate reader.

### Statistical analyses

Data of the DLR and the multiplex viability assays were processed and illustrated in GraphPad Prism 8 (GraphPad Software, La Jolla, USA). Study design to determine EC_x_ concentrations was conducted according to guidelines and recommendations (Green et al. 2018; Musset 2018), with at least six exposure concentrations in triplicates. However, initial screening experiments with BNF were conducted as a NOEC/LOEC analysis.

In terms of the NOEC/LOEC analyses, for both approaches (DLR and viability), mean background values (blanks) were subtracted from the raw values, and the data was computed as fold induction in relation to the negative controls. Means of every experiment were pooled (experimental unit n = 3–5; observational unit N = 9–15). Normality was tested by Shapiro–Wilk and Kolmogorov–Smirnov tests (both significance level alpha = 0.05) and analyzed graphically via normal-qq-plot. Non-normal data were log-transformed and re-analyzed. Given normality, statistically differing significance in the output signal from the negative controls was assessed via one-way ANOVA, followed by Dunnett’s post hoc test (for multiple comparisons vs. control). Residuals were graphically analyzed by quantile–quantile plot (actual vs. predicted residuals), homoscedasticity plot (absolute residuals vs. fitted), and residual plot (residuals vs. fitted) to ensure ANOVA criteria were met. Further, Brown–Forsythe and Bartlett’s tests were conducted to check for the residuals’ homoscedasticity and normality. Beyond statistical significance, for all viability tests, a threshold of 80% as compared to the negative control (corresponding 0.8) was determined as biologically significant and marked with a dotted red line within all respective graphs.

In terms of the EC_x_ (effect concentrations) and IC_x_ (inhibitory concentrations) regression analyses, DLR data was either assessed as relative induction or normalized induction (100 = max. overall fold response; 0 = min. overall fold response), given that normalized data results in overall better regression fits but is unable to display differences in efficacy. For EC_50_ and EC_20_ estimation, data were fitted to a four-parameter log-logistic (4PL) model with alternations described by (Weimer et al. 2012). Hence, curves were not fitted to upper and lower boundaries, but the controls were included in the regression as anchor values. Non-monotonous concentration–response curves (NMCRCs) were fitted likewise, using a bell-shaped nonlinear regression (as documented in https://www.graphpad.com/guides/prism/8/curve-fitting/reg_bellshaped_dose_response.htm).

Overall effects of the transfection setup on the recorded signal were analyzed via two-way ANOVA followed by Tukey’s post hoc test for multiple comparisons, with exposure concentrations and transfection setups both used as factors. An assessment of residuals was conducted, as stated above.

## Results

### Screening experiments of co-transfected normalization vectors

**Fig. 1:**
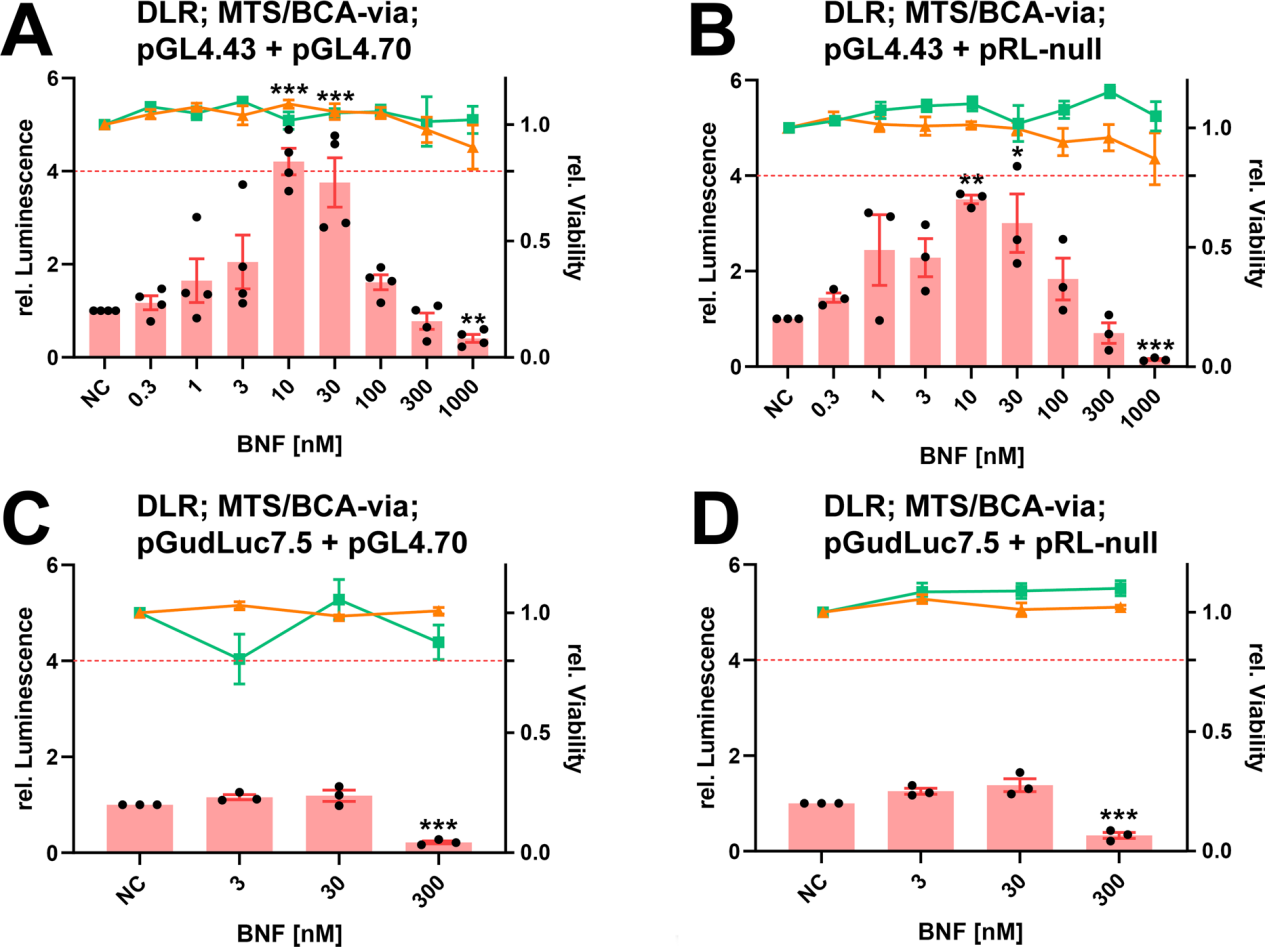
Effects on luminescence measured in the zebrafish cell line ZFL exposed to BNF. Luminescence corresponds to quantitative AhR transcription factor activation, measured via DLR assay in cells co-transfected with the depicted combinations of reporter and normalization vectors (**A**–**D**; all non-viral promoters). Mean normalized luminescence induction is illustrated as red bars, black dots represent means of single experiments, and red whiskers represent the SEM (experimental units n = 3–4; observational units N = 9–12). Cellular viability corresponds to apical endpoints measured via the MTS/BCA-multiplex assay. Each point (MTS orange, BCA green) represents the mean, including SEM (experimental units n = 3–4; observational units N = 9–12). A threshold value of 0.8 was considered biologically significant (dotted red line). Asterisks indicate significance tested in a one-way ANOVA with Dunnett’s post hoc test (*P < 0.05, **P < 0.01, ***P < 0.001)

**Fig. 2:**
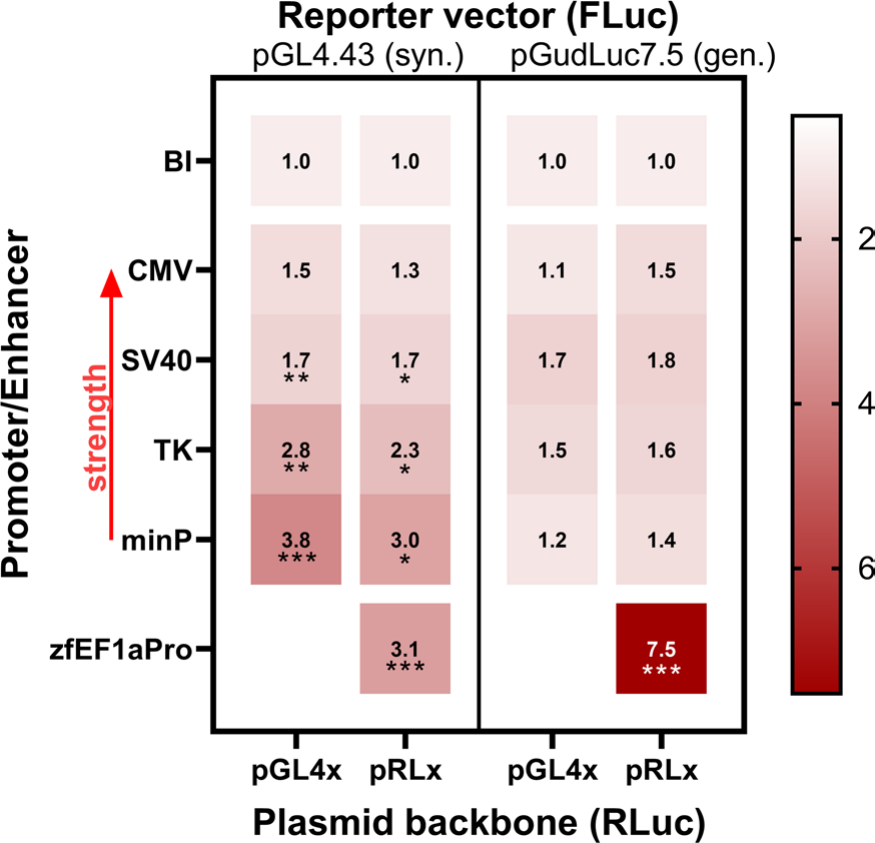
A heatmap summarizing AhR-activation related fold induction of all screening experiments at a 30-nM BNF exposure concentration. A total of 16 experimental transient co-transfection combinations has been conducted. The primary Firefly luciferase (FLuc) reporter vectors pGL4.43 (synthetic response element) and pGudLuc7.5 (genomic response element) were co-transfected with 8 Renilla luciferase (RLuc) normalization vectors of the pGL4 and pRL backbone series. The normalization vectors are bearing constitutive promoters of increasing strength (CMV > SV40 > TK > minP; red arrow), driving background RLuc expression. Maximum relative fold induction is given in relation to negative controls (base level induction, BI). Numerical fold induction is given for every combination. Transient co-transfection results are given for the respective pRL-null[zfEF1aPro] combinations, as well. Asterisks indicate significance tested in a one-way ANOVA with Dunnett’s post hoc test (vs. control BI; *P < 0.05, **P < 0.01, ***P < 0.001)

**Fig. 3:**
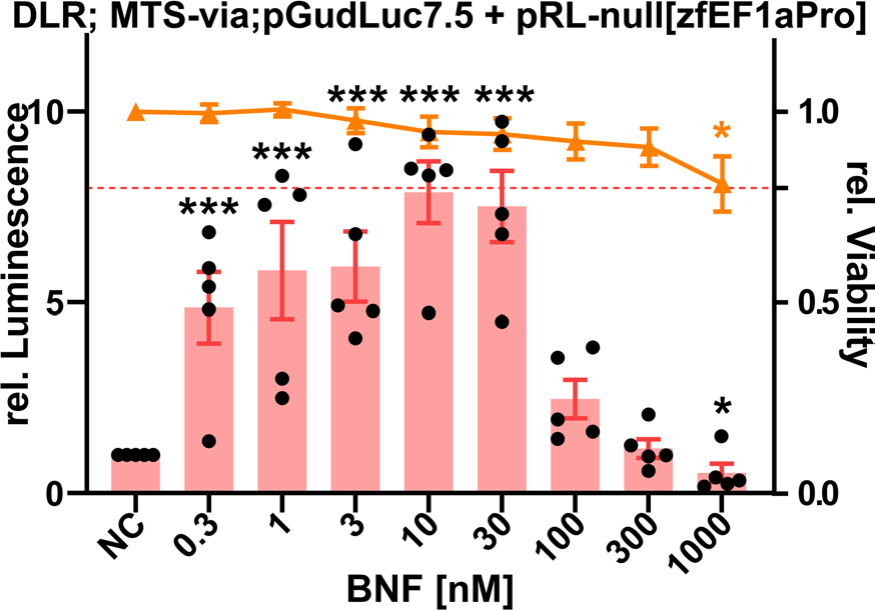
Effects on luminescence measured in the zebrafish cell line ZFL exposed to BNF. Luminescence corresponds to quantitative AhR transcription factor activation measured via DLR assay in cells co-transfected with the **pGudluc7.5** reporter and **pRL-null[zfEF1aPro]** normalization vector. Mean normalized luminescence induction is illustrated as red bars, black dots represent means of single experiments, and red whiskers represent the SEM (experimental units n = 5; observational units N = 15). Cellular viability corresponds to formazan turnover in the MTS viability assay. Each orange point represents the mean, including SEM (experimental units n = 5; observational units N = 15). A threshold value of 0.8 was considered biologically significant (dotted red line). Asterisks indicate significance tested in a one-way ANOVA with Dunnett’s post hoc test (*P < 0.05, **P < 0.01, ***P < 0.001)

In accordance with a previous study (Lungu-Mitea and Lundqvist 2020), both primary Firefly luciferase bearing reporter vectors were tested in transient co-transfection with all acquired normalization vectors. The normalization vectors were of two variations, consisting of either a **pGL4** or **pRL** backbone (Table 1, Fig. S2). Additionally, transcription of the Renilla luciferase normalization signal was either sequestered by a viral promoter (SV40, TK, CMV) or a minimal promoter construct (minP) on the respective backbone. Hence, eight normalization vectors were tested in the initial screening experiments. For the reporter vectors, two different constructs were tested. **pGL4.43** bears a synthetic promoter consisting of three repeat copies of the XRE core consensus sequence, fused to minP. **pGudLuc7.5** carries a genomic promoter of the *mCYP1A1* gene consisting of twenty copies of the mouse XRE sequence linked to the MMTV promoter (Han et al. 2004; He et al. 2011). Accordingly, a total of sixteen experimental setups was conducted within the first screening phase (Figs. 1, 2, S3, and S4). Cells transiently co-transfected with the specific setups were exposed to three increasing concentrations of BNF. Promising co-transfection setups were further tested in an entire concentration–response exposure regime (300 pM to 1 µM) (Figs. 1, 3, and S5). BNF was initially chosen as an agonist in the screening experiments due to less complicated handling and lower occupational hazard. Cellular viability was tested in parallel on identically co-transfected cells via the MTS/BCA-multiplex assay.

Appropriate AhR-related signal induction was only recorded for the **pGL4.43** reporter in combination with both normalization vectors that did not bear any viral promoters (Figs. 1A + B and 2). For the **pGL4.43 + pGL4.70** combination (Fig. 1A), the LOEC and the highest fold-induction of 4.1 were recorded at 10 nM. For the **pGL4.43 + pRL-null** combination (Fig. 1B), the LOEC and highest fold-induction of 3.9 was also recorded at 10 nM. Interestingly, BNF exposure did not induce an increasing or sigmoidal concentration–response curve. Instead, after AhR-related signal activation is peaking at around 10–30 nM, signal inhibition is encountered beyond 30 nM of BNF. The highest exposure concentration of 1 µM BNF led to statistically significant inhibition of the AhR-related signal in both co-transfection combinations. However, no statistically significant cytotoxicity was recorded for any of the apical endpoints tested within the entire concentration range (Fig. 1). Arguably, a slight trend in decline was detectable for the MTS endpoint beyond 300 nM BNF (Fig. 1A + B). Co-transfection with **pGudluc7.5** resulted in no overall statistically significant induction of the Ahr-related luciferase signal (Figs. 1C + D, 2, and S4). Nevertheless, the 300 nM BNF exposure concentration caused a statistically significant inhibition of the AhR-related signal for the non-viral promoter combinations (Fig. 1C + D) as well. Once more, no statistical significant cytotoxicity was recorded.

The **pGL4.43** vector also showed statistically significant induction in co-transfection with normalization vectors bearing viral promoters (Figs. 2 and S3). Statistically significant induction of the AhR-related luciferase response was recorded for the co-transfection combination **pGL4.43 + pGL4.74** (Fig. S3A), **pGL4.43 + pRL-SV40** (Fig. S3D), and **pGL4.43 + pGL4.73** (Fig. S3C). However, the induction was only within a 1.5- to threefold scale. Plasmids bearing TK-promoters also showed a downregulation of the signal in the highest exposure concentration (Fig. S3A + B). No statistically significant cytotoxicity was recorded for **pGL4.43** in combination with any viral promoter-bearing vector. However, **pRL-SV40** and **pRL-CMV** depicted cytotoxicity by biological threshold definition within the highest exposure concentration for at least one endpoint of cytotoxicity (Fig. S3D + F). The **pGudluc7.5** vector showed no significant statistical induction for any viral promoter-bearing normalization vectors in co-transfection (Figs. 2 and S4). Tentatively, an inhibition is detectable for TK and SV40-bearing vectors within the highest exposure concentrations (Fig. S4A-D). Cytotoxicity was recorded for CMV and SV40-bearing vectors within the highest exposure concentration, at least within one apical endpoint, in terms of either statistical or biological significance (Fig. S4C-F).

### The endogenous zfEF1a promoter restores AhR-related luciferase induction after BNF exposure

Given that only normalization vectors without viral promoters resulted in an appropriate signal induction, **pRL-null** was chosen as a template for directional cloning of the endogenous zebrafish translation elongation factor 1 alpha genomic promoter (zfEF1aPro). The **pRL-null** multiple cloning site (MCS) exhibited a priori the correct topography to insert zfEF1aPro in the forward direction, whereas **pGL4.70** would have required additional assembly PCRs. Subsequently, the newly cloned **pRL-null[zfEF1aPro]** normalization vector (Fig. S1) was co-transfected with both used reporter vectors. The **pGL4.43 + pRL-null[zfEF1aPro]** co-transfection was almost identical to **pGL4.43 + pRL-null**, with the former depicting a slightly lower LOEC (3 nM) and an identical maximum induction of 3.9-fold at a concentration of 10 nM (Fig. S5). However, increased LOEC sensitivity might derive from the higher amount of replicates used in the statistical test (*n* = 5 vs. *n* = 3). Nevertheless, the **pGudluc7.5 + pRL-null[zfEF1aPro]** co-transfection setup showed significant differences from the **pGudluc7.5 + pRL-null** setup. Integration of the genomic zfEF1a promoter restored the BNF-induced luminescence signal (Figs. 2 and 3). Maximal induction almost doubled to 7.9-fold (10 nM BNF), and the LOEC was reduced to 300 pM. Arguably, a more sensitive LOEC could have been measured if the exposure regime were accordingly adapted. In accordance with the screening experiments, concentrations beyond 30 nM BNF induced inhibition of the AhR-related luminescence signal. Statistically significant cytotoxicity was recorded at a concentration of 1 µM BNF. Here, only the MTS endpoint was measured since the screening experiments depicted no systemic difference between the MTS and BCA endpoints.

**Fig. 4:**
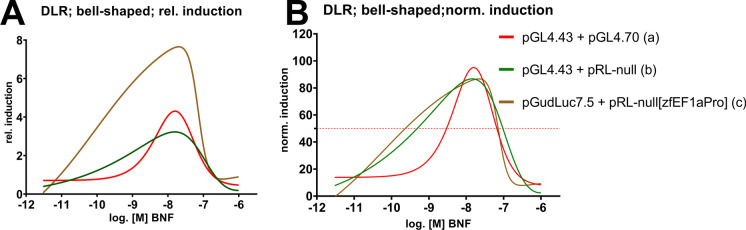
Bell-shaped concentration–response curves of depicted co-transfection setups after BNF exposure. Results of the DLR assays were either fitted as relative induction (**A**) or normalized induction values (**B**) to the nonlinear regression. EC_50_, IC_50_, and PC_max_ values are summarized in Table 2. Specific concentration–response curves are illustrated in Fig. S6

The data of the most advantageous co-transfection setups were additionally fitted to bell-shaped concentration–response nonlinear regressions, with alternations according to (Weimer et al. 2012) (Fig. 4). EC_50_ (50% effective concentration), IC_50_ (50% inhibitory concentration), and PC_max_ (maximum peak concentrations) were respectively derived from the regressions (Table 2). The nonlinear regressions were either computed with the relative induction data (Fig. 4A), as also depicted in the previous graphs (Figs. 1 and 3), or with normalized induction data (0–100% response, Fig. 4B). Normalized data often results in better fits (see parameters of fit, adjusted R^2^ and NRSME values in Table 2) but is not suited for displaying differences in efficacy. Overall, the **pGudluc7.5 + pRL-null[zfEF1aPro]** co-transfection setup depicted the highest efficacy with the relative induction of the luciferase signal plateauing at 7.9-fold (Fig. 4A). At the same time, the **pGL4.43 + pGL4.70** and **pGL4.43 + pRL-null** co-transfection setups showed similar plateaus in the 3.5 to 4.2-fold induction range. Interestingly, the **pGudluc7.5 + pRL-null[zfEF1aPro] (EC**_**50**_** 63 pM)** and **pGudluc7.5 + pRL-null (EC**_**50**_** 490 pM)** setups depicted gentler slopes than **pGL4.43 + pGL4.70 (EC**_**50**_** 3 nM)**, thus also showing an increased potency to the BNF exposure. PC_max_ were per se similar, ranging from 15.7 to 19.5 nM, indicating a common peak concentration, regardless of the co-transfection setup.The IC_50_ values were all in the same range, approximately 20–100 nM, regarding the regression model used. Hence, the inhibition in higher BNF concentrations is not affected by the co-transfection setup used but derives from another common systemic mechanism. Comparison of overall mean co-transfection setup effects resulted in no statistical differences for the normalized data. Still, the **pGudluc7.5 + pRL-null[zfEF1aPro]** combination is statistically differing from the other two in terms of relative induction (Table 3). Single bell-shaped concentration–response curves, including confidence intervals, means, and SEMs, are depicted in the SI (Fig. S6).

**Table 2:** LOEC, EC_50_/IC_50,_ and PC_max_ values derived from BNF exposure (adj. R^2^ and NRSME values are given as goodness-of-fit parameters of the nonlinear regression)

Transfection setup (according to Fig. 4)		LOEC (adj. *P*-value)	EC_50_ (normalized; adj. R^2^; NRSME)	IC_50_ (normalized; adj. R^2^; NRSME)	EC_50_ (relative; non-normalized; adj. R^2^; NRSME)	IC_50_ (relative; non-normalized; adj. R^2^; NRSME)	PC_max_ (relative, non-normalized)	N (exp.)
(a)	pGL4.43 + pGL4.70	10 nM (< 0.0001)	2.95 nM (0.8; 0.39)	24 nM (0.80; 0.32)	2.45 nM (0.77; 0.02)	27 nM (0.77; 0.02)	15.8 nM (0.77; 0.02)	4
(b)	pGL4.43 + pRL-null	10 nM (0.0044)	490 pM (0.71; 0.71)	106 nM (0.72; 0.55)	309 pM (0.72; 0.03)	109 nM (0.72; 0.02)	15.7 nM (0.72; 0.02	3
(c)	pGudLuc7.5 + pRL-null[zfEFapro]	300 pM (0.003)	63 pM (0.79; 0.31)	73 nM (0.79; 0.31)	87 pM (0.75; 0.03)	72 nM (0.75; 0.03)	19.5 nM (0.75; 0.03)	5

**Table 3:** Comparison of mean co-transfection setup effects between combinations depicted in Fig. 4 and Table 2. Statistical significance in mean setup effect was assessed via 2-way ANOVA, followed by Tukey**’**s post hoc test (ns *P* > 0.05, ***P < 0.001)

Comparison	Adjusted *P*-value	Significance
a vs. b (rel.; Fig. 4A)	0.8146	ns
a vs. c (rel.; Fig. 4A)	< 0.0001	***
b vs. c (rel.; Fig. 4A)	< 0.0001	***
a vs. b (norm.; Fig. 4B)	0.9359	ns
a vs. c (norm.; Fig. 4B)	0.9936	ns
b vs. c (norm.; Fig. 4B)	0.9623	ns

### Efficacy and potency of TCDD exposure in specific co-transfection setups

**Fig. 5:**
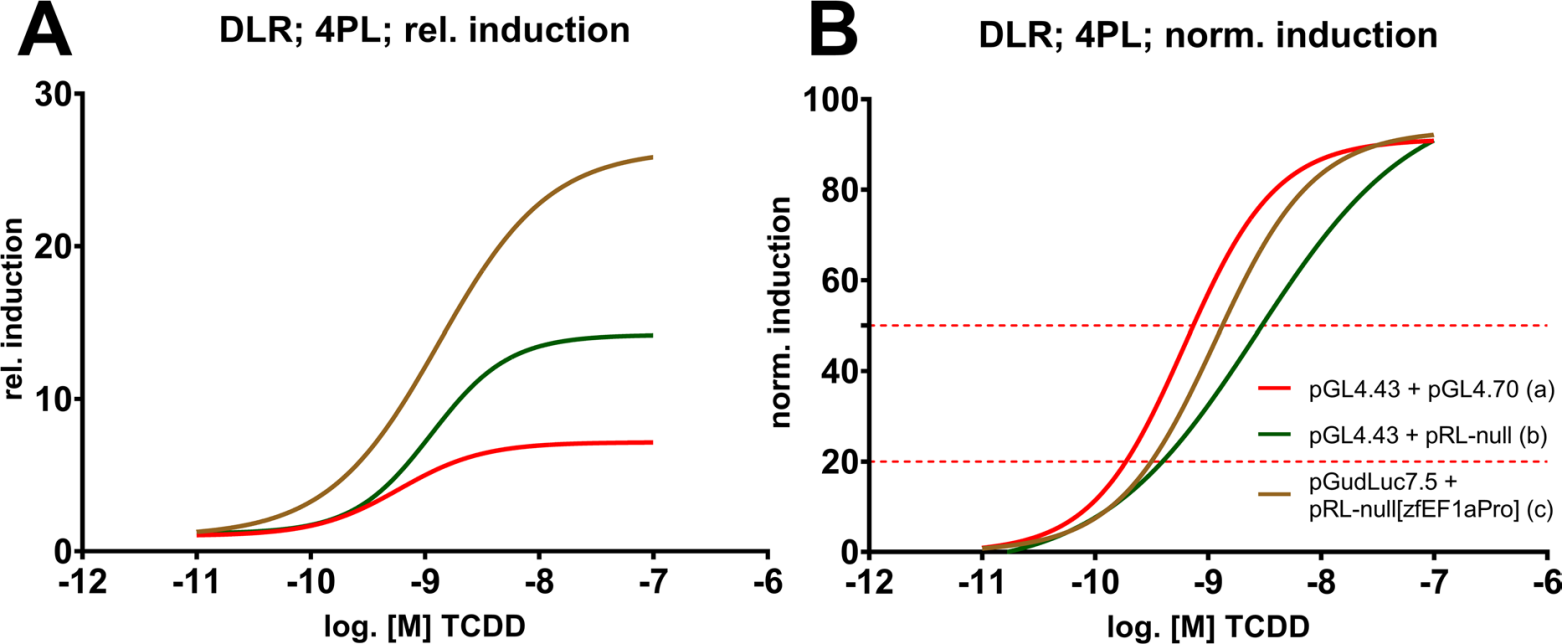
Concentration–response curves of depicted co-transfection setups after TCDD exposure. Results of the DLR assays were either fitted as relative induction (**A**) or normalized induction values (**B**) to a four-parameter log-logistic nonlinear regression (4PL). EC_20/50_ values are summarized in Table 4. Specific concentration–response curves are illustrated in Fig. S8

All non-viral promoter setups were further tested in DLR assays after TCDD exposure, and data were fitted to nonlinear four-parameter log-logistic regression (4PL) with alterations described by (Weimer et al. 2012). In parallel to data of the BNF exposures above, both relative and normalized data outputs were computed and plotted. Concentration–response curves of the **pGL4.43 + pGL4.70**, **pGL4.43 + pRL-null**, and **pGudluc7.5 + pRL-null[zfEF1aPro]** co-transfection setups are depicted below (Fig. 5), all other conducted test are shown in the SI (Fig. S8 and Tables S1 + 2). Initially, responses to TCDD exposure were tested since it is commonly used as a reference/positive control in toxicity testing, especially for deriving toxicity equivalents. In terms of efficacy, patterns were identical to the BNF exposure, with the **pGudluc7.5 + pRL-null[zfEF1aPro]** combination depicting the most potent induction and plateauing at approximately 26-fold (Fig. 5A). Accordingly, the mean co-transfection setup effect differed statistically between all tested combinations (Table 5). Surprisingly, the **pGL4.43 + pGL4.70** depicted the highest potency (Fig. 5B and Table 4), differing statistically from **pGL4.43 + pRL-null** in terms of the overall effect of the normalized response (Table 5). Nevertheless, EC_50_ values (Table 4) differed only by approximately twofold and EC_20_ values were all recorded within the range of 200 pM. Further, these statements also account for all the other tested combinations, as depicted in the SI (Fig. S8 and Tables S1 + 2). Arguably, slight differences between co-transfection setups might rather be artefacts from manual titration and suboptimal nonlinear regression model-fit for some combinations. Thus, in terms of the TCDD exposure, using the **pGudluc7.5 + pRL-null[zfEF1aPro]** co-transfection setup only increased the efficacy of the applied method but not the potency. In hindsight, directional cloning of the zfEF1a promoter into the **pGL4.70** normalization vector would have potentially added more information, given that after TCDD exposure, pGL4.70 vectors showed increased potency in co-transfection setups in comparison to **pRL-null** vectors (Table S1). Cytotoxicity was only recorded for the **pGudluc7.5 + pRL-null[zfEF1aPro]** combination, which displayed none (Fig. S7). Further assessments of cellular viability were waived given that the non-viral promoter normalization vectors showed no additional cytotoxicity in the screening experiments and historical data displayed no TCDD-induced cytotoxicity within the same cellular context and within identical exposure concentrations (Eknefelt 2018).

**Table 4:** LOEC and EC_20/50_ values derived from TCDD exposure (adj. R^2^ and NRSME values are given as goodness-of-fit parameters of the nonlinear regression). According to setups depicted in Fig. 5

Transfection setup (according to Fig. 4)		LOEC (adj. *P*-value)	EC_20_ (normalized; adj. R^2^; NRSME)	EC_50_ (normalized; adj. R^2^; NRSME)	EC_50_ (relative; non-normalized; adj R^2^; NRSME)	N (exp.)
(a)	pGL4.43 + pGL4.70	100 pM (0.0406)	166 pM (0.88; 0.19)	612 pM (0.88; 0.19)	595 pM (0.57; 0.29)	8
(b)	pGL4.43 + pRL-null	300 pM (0.008)	297 pM (0.85; 0.28)	2.70 nM (0.85; 0.28)	1.31 nM (0.47; 0.11)	6
(c)	pGudLuc7.5 + pRL-null[zfEF1aPro]	100 pM (0.0003)	287 pM (0.92; 0.16)	1.15 nM (0.92; 0.16)	1.13 nM (0.62; 0.11)	8

**Table 5:** Comparison of mean co-transfection setup effects between combinations depicted in Fig. 5 and Table 4. Statistical significance in mean setup effects was assessed via two-way ANOVA, followed by Tukey**’**s post hoc test (ns *P* > 0.05, **P < 0.01, ***P < 0.001)

Comparison	Adjusted *P*-value	Significance
a vs. b (rel.; Fig. 5A)	0.0015	**
a vs. c (rel.; Fig. 5A)	< 0.0001	***
b vs. c (rel.; Fig. 5A)	< 0.0001	***
a vs. b (norm.; Fig. 5B)	0.0025	**
a vs. c (norm.; Fig. 5B)	0.2858	ns
b vs. c (norm.; Fig. 5B)	0.4350	ns

### BNF acts as a promiscuous activator of cellular stress response pathways

ZFL cells were also transiently co-transfected with the Nrf2-responsive reporter vector pGL4.37 and the normalization vector pRL-CMV, as described in (Lungu-Mitea and Lundqvist 2020). The transcription factor Nrf2 is a central regulator of the oxidative stress response pathways (Nrf2/Keap1/ARE) (Itoh et al. 2004) and a keystone in the regulation of oxidative stress detoxification, metabolism, and induction of related phase-II enzymes (e.g., GST, NQO1, HO-1). Detailed descriptions, results, and discussion are given in the respective section of the SI (p. 12–14; Figs. S10 + 11, Table S4). We recorded a LOEC of 30 nM and an EC_50_ of 9.7 nM within a BNF exposure range of 3 pM to 1 mM.

## Discussion

The principal aim of this study was to develop a transient reporter gene assay of the AhR xenobiotic metabolism pathway in zebrafish hepatocytes, robust enough to be used in environmental testing. Within the introduction, we mentioned the potentials inherent to transient assays but also indicated issues that accompany transient transfection and might lead to artificial results (Stepanenko and Heng 2017; Lungu-Mitea and Lundqvist 2020). Thus, the study’s secondary aim was to disclose the origins of spurious expression or artificial signal inhibition that might originate from specific vector geometries. Zebrafish cells were chosen as a test model considering the zebrafish’s progressively important role in toxicology, both in basic research and in testing (Garcia et al. 2016). Further, all xenobiotic metabolism pathway (AhR/ARNT/XRE) components are well described in zebrafish (Tanguay et al. 1999, 2000; Andreasen et al. 2002; Zeruth and Pollenz 2005, 2007; Hahn et al. 2017). The zebrafish liver cell line (ZFL) seemed an appropriate host for transfection of transient reporter vectors of the xenobiotic metabolism pathway, given that former research confirmed the presence of major components of the pathway in the permanent, immortal culture. Reports (Miranda et al. 1993; Ghosh and Collodi 1994; Ghosh et al. 1994) disclosed the activity of phase I xenobiotic metabolism enzymes of the cytochrome P450 (CYP) family on the proteomic level. Others (Henry et al. 2001; Evans et al. 2005; Eide et al. 2014) reported basal activity and induction capacity of the *zfAhR2, zfAhRR,* and *zfCyp1A1* genes on the transcriptomic level. Beyond, one stable GFP-reporter (Mattingly et al. 2001) and one stable luciferase-reporter cell line (Yang et al. 2016; Chen and Chan 2017; Zhou et al. 2017) were derived from ZFL, demonstrating conservation and functionality of the AhR/ARNT/XRE xenobiotic metabolism response pathway.

### Viral promoters squelch the AhR-related reporter signal in ZFL cells and induce size-dependent cytotoxicity

**Fig. 6:**
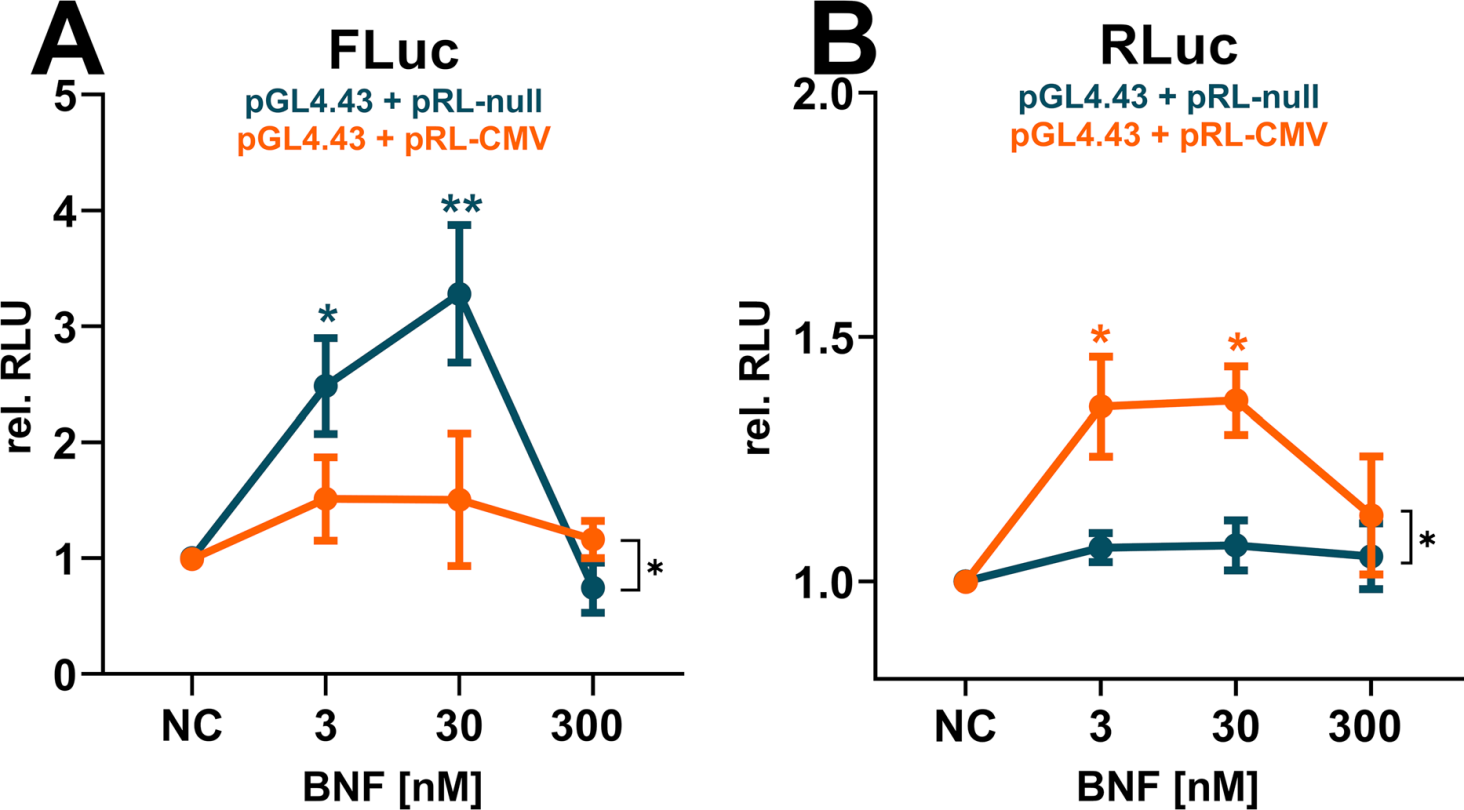
Effects on relative raw luminescence units (rel. RLU) in the ZFL cell line exposed to BNF. Here, single luminescence signals are depicted: Firefly luciferase (FLuc) is the primary reporter signal derived from the expression of the reporter vector (**A**); Renilla luciferase (RLuc) is the normalization signal derived from the expression of the control vector (**B**). Mean relative RLU induction is illustrated as dots with whiskers, including the SEM (experimental units n = 3–5; observational units N = 9–15). Colored asterisks indicate significance tested in a one-way ANOVA with Dunnett’s post hoc test (*P < 0.05, **P < 0.01) within a specific co-transfection setup. Black asterisks indicate significance tested in a two-way ANOVA with Tukey’s post hoc test (*P < 0.05) to detect overall effects between co-transfection setups

The primary results of the BNF-exposure screening on all sixteen tested co-transfection combinations revealed appropriate induction of the AhR-related signal only for the synthetic reporter construct on **pGL4.43** combined with minimal promoter bearing normalization vectors (Figs. 1, 2, S3, and S4). All co-transfection combinations utilizing viral promoters showed minimal induction of the AhR-related luminescence or none at all. Thereby, the reduced signal inductivity was correlated to the strength of the specific viral promoters (CMV > SV40 > TK; Fig. 2), as it was most apparent for the pRL-CMV vector, with a spurious upregulation of the Renilla luciferase (RLuc) normalization signal (Fig. 6B), leading to a downregulation of the primary Firefly luciferase (FLuc) reporter signal (Fig. 6A). The pattern was not statistically significant for weaker viral promoters (data not plotted) or for any of the non-viral promoter co-transfection combinations involving the **pGudLuc7.5** reporter vectors. However, they were cognizable. Previously, we reported the conservation of viral promoter strength and associated transcription factors in the ZFL cell line (Lungu-Mitea and Lundqvist 2020). Nevertheless, the recorded luciferase signal has to be regarded in an integrative manner. Hence, an alteration of the reporter signal is not necessarily recordable by normalization signal upregulation. The phenomenon is defined as transcriptional “squelching” (Natesan et al. 1997), which refers to the competition between gene regulatory units for transcription factors, co-activators, and the overall transcription/translation machinery (Martino et al. 2004; Simon et al. 2015). Accordingly, viral promoter induced squelching has been reported for several cell lines, transfection constructs, and studied pathways (reviewed in Shifera and Hardin 2010; Stepanenko and Heng 2017)). Thereby, the effect cannot be generalized but depends on the utilized cellular context, the used transfection methods, the specific plasmid vector geometry, and the pathway studied (Mulholland et al. 2004; Lungu-Mitea and Lundqvist 2020). Correspondingly, we found the **pRL-CMV** normalization vector most potent in a co-transfection setup for assessing the induction of the Nrf2 cellular stress response pathway (Lungu-Mitea and Lundqvist 2020).

Except for the TK promoter, we once more (Lungu-Mitea and Lundqvist 2020) recorded a vector size-dependent increase in cytotoxicity when combining transient transfection with chemical exposure. SV40 and CMV-bearing vectors caused additional cytotoxicity compared to minP-containing vectors (Figs. S3 + 4; see Table 1 for vector size). Previous reports disclosed the vector-size dependent induction of the cellular immune response during transfection experiments (Jacobsen et al. 2009) and its interaction with the perceived signal (Ghazawi et al. 2005) by mimicking a viral infection due to the existence of exogenous, episomal genetic material (Terenzi et al. 1999). Such an induction will accelerate the inflammatory response and, thus, cytotoxicity. Interestingly, this effect was inverted by using the endogenous genomic zfEF1a promoter (Fig. 2).

### Modulation of the luciferase reporter signal in transiently transfected ZFL cells after BNF and TCDD exposure

Except for the **pGudLuc7.5 + pRL-null[zfEFapro]** co-transfection setup, which will be discussed in more detail further below, TCDD was the stronger AhR-agonist within all tested arrangements in comparison to BNF (see Tables 2 and 4). The latter statement is according to the literature, with the AhR showing greater affinity to the TCDD ligand in comparison to BNF (Soshilov and Denison 2014; Tagliabue et al. 2019). However, the ZFL system seems relatively insensitive to TCDD exposure with EC_20_s computed in the 200 pM range and EC_50_s in the 612 pM to 2.7 nM range, in regard to the co-transfection setup used (Tables 4 and S1). Mammalian reporter assays, especially rodent-derived cell lines, depict EC_50_s in the low pM range and limits of detection even within the fM range (Sanderson et al. 1996; Eichbaum et al. 2014). Apparently, mammalian-based assays are more sensitive to TCDD (rodent > human) and other dioxin-like compounds than fish-based assays (rainbow trout > zebrafish), due to structural differences and affinities of their respectively recruited AhRs (Abnet et al. 1999; Hilscherova et al. 2001; Keiter et al. 2008; Eichbaum et al. 2014). Even within fish, zebrafish is the most insensitive, commonly used test species, as demonstrated in vivo (Elonen et al. 1998; Jönsson et al. 2009; Doering et al. 2012) and in vitro (Creusot et al. 2014). Hence, the regulatory relevance of zebrafish-derived reporter assays of the AhR-regulated xenobiotic metabolism TP is in dispute. On the contrary, the system shows great potential in basic research and in vitro to in vivo extrapolation (IVIVE), given the wide acceptance of zebrafish in in vivo studies and the zebrafish embryo test (FET) (Braunbeck et al. 2005; Belanger et al. 2013).

Interestingly, we recorded a non-monotonous, bell-shaped (or bi-phasic) concentration–response curve (NMCRC) after BNF exposure. Thereby, the inhibition was not dependent on the co-transfection setup, with all tested setups showing approximate PC_max_s (15.7–19.5 nM) and IC_50_s (27–109 nM, see Table 2). Hence, a common, non-transfection related mechanism might be responsible for the downregulation, and cytotoxicity has been ruled out. The AhR is promiscuous and binds to various ligand classes with different affinities (Soshilov and Denison 2014). Other classes of ligands might also alternate translocator recruitment. Within the AhR/ARNT/XRE pathway, upon activation by specific classes of ligands, AhR can dimerize with alternating co-factors to act as a transcription factor in non-canonical pathways (reviewed in Denison and Faber 2017; Wright et al. 2017)).

Josyula et al. (2020) mapped the entire AhR-mediated transcriptional regulatory network and identified many endogenous functions such as lipid metabolism, cellular immune response, and cell migration. Further, they found direct and combinatorial control (“tethering”) of gene expression of various cellular stress response pathways by AhR. TPs of the xenobiotic metabolism (PXR, PPAR, AhR), the hormone response (ER, AR, GR), and the cellular stress response (Nrf2, p53, HIF-1, NfkB, MTF, among others) are known to exhibit crosstalk within their specific domains due to a commonly shared structural architecture (reviewed in Simmons et al. 2009; Lushchak 2011)). However, an increasing body of literature indicates the existence of generalized crosstalk between all primary TPs. Among others, AhR has been found to interact with the ER (Safe 1995; Safe et al. 1998; Klinge et al. 2000; Ohtake et al. 2003; Cheshenko et al. 2006), Nrf2 (Ma et al. 2004; Miao et al. 2005; Köhle and Bock 2007; Shin et al. 2007; Yeager et al. 2009; Raghunath et al. 2018), and NfkB (Schlezinger et al. 2000a; Tian 2009) TPs. Additionally, a close structural architecture to the hypoxia-inducible factor 1 (HIF1) pathway (Schmidt and Bradfield 1996; Andreasen et al. 2002; Simmons et al. 2009) locates AhR close to the stress response pathways. Regarding cellular stress response pathways, the transcription factors are mostly pathway-specific; however, transducers and translocators are often shared, enabling a rapid response to stressors by mounting the cellular defence mechanisms on multiple ends (Simmons et al. 2009; Lushchak 2011). Such a multiple upregulation may either be encountered at once, close to concentrations causing cytotoxicity (termed “avalanche effect” or “toxicity burst” (Escher et al. 2013; Judson et al. 2016)), or in an orchestral manner with one stressor activating different pathways at different effect concentrations (Simmons et al. 2009; Lushchak 2011).

**Fig. 7:**
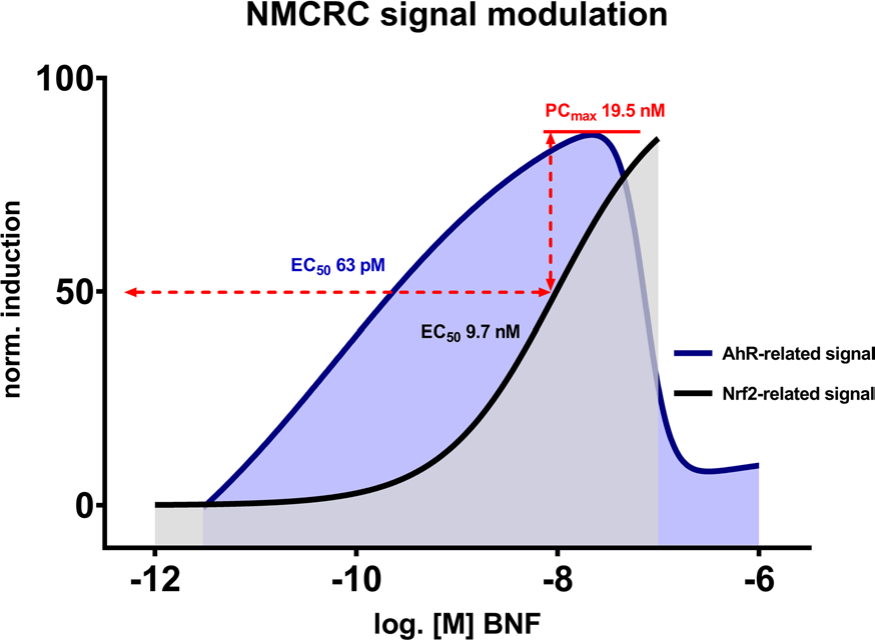
Schematic illustration of BNF-induced CRCs in a xenobiotic metabolism (AhR) and oxidative stress (Nrf2)-related transient reporter gene assay in ZFL cells. Hypothetically, NMCRCs, as recorded for the AhR-related signal, are due to TPs squelching or feedback loop mechanisms, given BNF’s promiscuity. Consult Figs. S6C and S11 for actual CRC-fits

We retrieved ToxCast database outputs via the CompTox dashboard (https://comptox.epa.gov/dashboard) for BNF and encountered positive hits for diverse TPs (Fig. S9 and Table S3) ahead of the cytotoxicity cutoff. Many of the major TPs of the xenobiotic metabolism (AhR, RAR, PPAR, PXR), hormone response (ER, AR, VDR), and cellular stress response (Nrf2) are activated by BNF exposure in the mid nM to lower µM scale. ER, AhR, and Nrf2-related bioassays depicted top-scale induction. Interestingly, the histone deacetylase inhibitor (HDACi) epigenetic marker showed a notable upregulation, indicating a general impact on gene regulation. Additionally, we decided to test the induction of the Nrf2 cellular stress response pathway in the ZFL cell line after exposure to BNF by employing a previously described DLR reporter gene assay (Lungu-Mitea and Lundqvist 2020). For more details, see the respective section in the SI (Figs. S10 + 11; Table S4). Interestingly, we saw maximal Nrf2 induction at 100 nM BNF (Fig. S10) coinciding with concentrations triggering an AhR inhibition (e.g., Figs. 1 and 3). Further, we computed an EC_50_ for Nrf2 induction at 9.7 nM (Fig. S11 and Table S4), which coincides with PC_max_s of the NMCRCs in the range of 15.7 to 19.5 nM. Taken together, BNF most likely acts as an inducer of multiple TPs. Figure 7 depicts how NMCRC might be modulated via squelching by BNF promiscuously activating multiple toxicity pathways.

Accordingly, NMCRCs were also recorded after BNF exposure in zebrafish embryos (Noury et al. 2006), adult guppy (Frasco and Guilhermino 2002), and rainbow trout hepatocytes (RTL-W1; (Heinrich et al. 2014)) but so far lacked explanation. Beyond, non-monotonicity was also encountered after exposure to various dioxin-like compounds (DLCs) and polycyclic aromatic hydrocarbons (PAHs) when recording CYP1A1 activity via the EROD-assay, in both in vivo and in vitro test systems, at non-cytotoxic concentrations, and in various species (Gooch et al. 1989; Hahn et al. 1993, 1996; Hahn and Chandran 1996; Verhallen et al. 1997; White et al. 1997; Delescluse et al. 1997; Tysklind et al. 1998; Schlezinger et al. 1999, 2000b, 2006; Wassenberg et al. 2005). Thereby, NMCRCs were limited to CYP1A1 activity recorded via the EROD-assay, whereas total CYP1A1 protein amounts (Hahn et al. 1993) and mRNA transcript levels (White et al. 1997; Delescluse et al. 1997) were either stable or increasing with increasing exposure concentrations. A few possible explanations of the phenomenon were proposed: inhibition of heme synthesis and urophyria (Hahn and Chandran 1996; Tysklind et al. 1998), competitive inhibition of the EROD enzyme–substrate reaction by DLCs and PAHs (Petrulis and Bunce 1999), or uncoupling of the CYP1A1 catalytic cycle by respective compounds resulting in ROS formation within the active site (White et al. 1997; Schlezinger et al. 1999, 2000b, 2006). Further, ROS were associated with a downregulation of overall CYP1A1 amount and activity (White et al. 1997; Xu and Pasco 1998; Schlezinger et al. 1999).

In this study, we saw NMCRCs after BNF but not after TCDD exposure. To our knowledge, there is no literature interpreting such a phenomenon for flavonoids or polyphenols, but similar mechanisms seem plausible due to structural proximities to DLCs and PAHs (abbreviations given in SI). Only sigmoidal concentration–response curves were recorded here after TCDD exposure. Theoretically, NMCRCs would also be encountered here if exposure regimes were appropriately extended. However, worth mentioning, maximal exposure concentrations in this study were operating close to the onset of TCDD-induced cytotoxicity in ZFL cell lines (Eknefelt 2018). Thus, the latter would have superimposed a potential NMCRC.

**Fig. 8:**
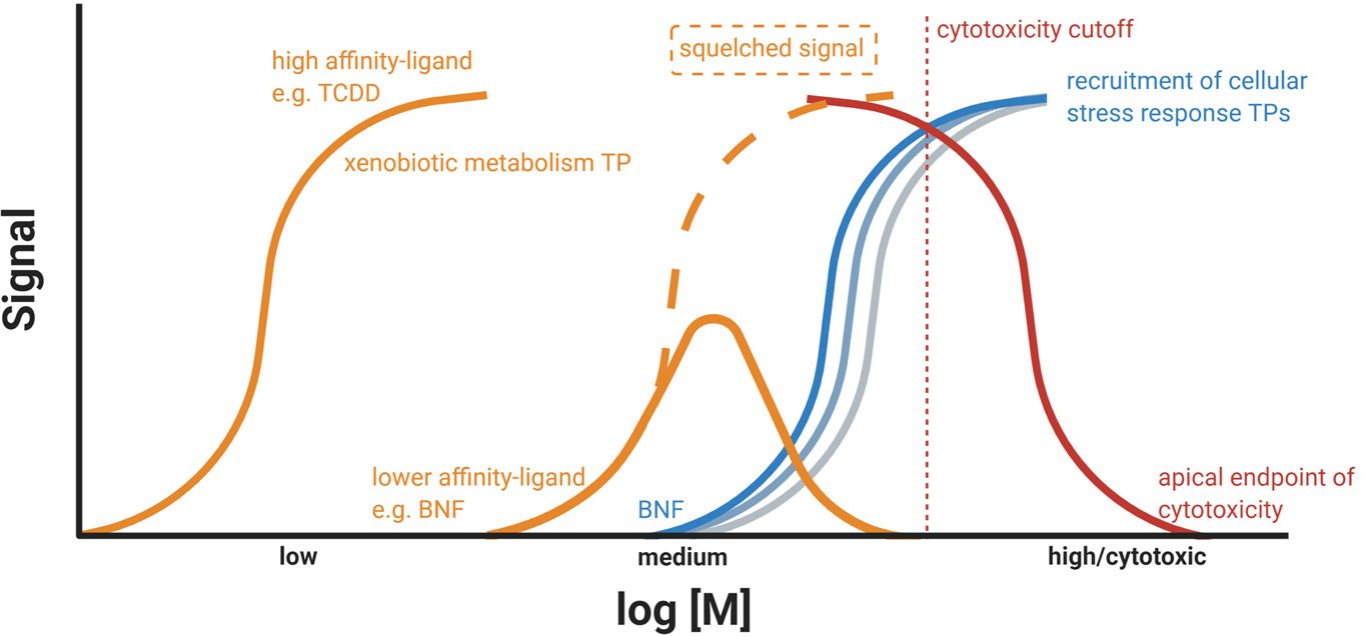
Schematic representation of the hypothetical squelching of the AhR TP-related luciferase signal (orange) due to activation of alternate cellular stress response pathways (shades of blue) in a system under increasing toxic stress, up to cytotoxicity (red). The illustration was generated in BioRender

Taken together, we cannot precisely define if downregulation of the AhR/ARNT/XRE pathway for NMCRCs materializes on the level of transactivation, transcription, or translation, as such was not the purpose of the study. Instead, we hypothesize a squelching phenomenon, given the promiscuous induction of TPs by BNF on the gene transactivation level, coinciding at specific EC_50_, IC_50_, and PC_max_ concentrations. The presented data adds crucial information on the appearance of NMCRCs after BNF exposure. However, further investigation will be necessary to pinpoint its core mechanism. Given the existence of generalized crosstalk between all primary TPs, as discussed above, it is, however, plausible that interactions originate on the level of transactivation. Hypothetically, negative feedback loops from uncoupling-derived ROS (White et al. 1997; Xu and Pasco 1998; Schlezinger et al. 1999) or alternate modulation of mRNA transcript stability (Zhao et al. 2021) are other vital explanations. We want to advocate here that the encountered NMCRCs might be a result of squelching between multiple TPs recruiting the transcription/translation machinery simultaneously (Fig. 8), as indicated by our data and ToxCast outputs. The transactivation of multiple TPs by single compounds has been rigorously described (Kamei et al. 1996; Xu and Pasco 1998; Klinge et al. 2000; Schlezinger et al. 2000a; Ma et al. 2004; Miao et al. 2005; Köhle and Bock 2007; Shin et al. 2007; Yeager et al. 2009; Kalthoff et al. 2010; Ulin et al. 2019). However, we cannot identify if BNF acts *a priori* as a multiple inducer or if an original signal is sequestered into numerous responses. Conclusively, recorded patterns are a linear, one-dimensional snapshot of a multi-dimensional feature.

### The zfEF1a genomic promoter rescues the luciferase signal from “maisonette squelching”

It is recommended to test reporter gene assays containing synthetic and genomic promoters to respectively account for differences in specificity and sensitivity (Simmons et al. 2009). In terms of reporter vectors, we utilized both types with **pGL4.43** (synthetic) and **pGudLuc7.5** (genomic). Further, we designed a novel normalization vector **pRL-null[zfEF1aPro]** by directional cloning of the zebrafish translational elongation factor 1a promoter, a highly active genomic promoter (Gao et al. 1997), into the multiple cloning site of the **pRL-null** plasmid. Reporter cassettes employing the EF1a promoter were reported to enable a robust and constant expression of desired episomal target genes and, beyond, were not prone to gene-silencing once applied in stable transfection (Gopalkrishnan et al. 1999; Wang et al. 2017). Above, we established how strong viral promoters squelched the luciferase signal (Figs. 2 + 6) and potentially introduced cytotoxicity via inflammation (Figs. S3 + S4). On the contrary, a genomic promoter is less likely to be considered exogenous material by the cellular immune response. It might be regulated natively by the cellular transcription/translation machinery, thus, not being prone to spurious up and downregulation. Beyond, viral promoters often bear cryptic binding sites, such as for the activator protein 1 (AP1) transcription factor (Kushner et al. 1994; Grimm and Nordeen 1999; Hall et al. 2002; Dougherty and Sanders 2005). The latter regulates manifold gene expression in the context of cellular metabolism and homeostasis, as it is associated with the MAPK pathways (Swanson et al. 1995; Shifera and Hardin 2009). Within a stressed system, AP1 will be alternatively regulated and impact the expression of episomal target genes on plasmid vectors fused to viral promoters. On the contrary, genomic EF1a promoter bear reduced cryptic binding sites compared to viral promoters (Wang et al. 2017), making them less susceptible to spurious regulation within a stressed system.

In this study, we encountered a complete rescue of the AhR-mediated signal when applying the **pGudLuc7.5 + pRL-null[zfEF1aPro]** co-transfection setup (Figs. 1, 2, and 3). Beyond, we recorded an overall increase in efficacy and partly in potency (Figs. 4 and 5) for the genomic reporter construct **pGudLuc7.5**. However, those enhancements were only marginally encountered when using the **pGL4.43 + pRL-null[zfEF1aPro]** co-transfection setup (Fig. S5). Apparently, by utilizing the zfEF1a genomic promoter, vector-mediated squelching based on strong viral promoters was omitted. Further, an inflammatory response of the cell line due to transfection was most likely avoided due to the genomic promoter’s endogenous nature. In parallel to our study, other studies confirmed ROS-mediated transcriptional suppression of the AhR/ARNT/XRE TP in dependency of the inducible transcriptional strength of XREs within their respective reporter gene cassettes (Xu and Pasco 1998). Thereby, conditions that increased the transactivation potential of AhR attenuated the action of ROS, whereas conditions that reduced XRE-mediated transactivation potentiated the inhibitory action of ROS. Identically, we saw a complete inhibition of transactivation for unbefitting co-transfection combinations (e.g., Fig. 1C).

In theory, we regard the more or less complete initial silencing of the **pGudLuc7.5** (Figs. 1 and 2) reporter vector as squelching on multiple levels: Firstly, on the level of plasmid-bound gene-regulatory units competing with each other and episomal target genes for the recruitment of transcription factors and co-factors (Fig. 6). Secondly, on the level of competing TPs for the same machinery as an overall detoxification response (Figs. 7 and 8). Prospectively, we want to declare and address such a multi-level squelching phenomenon, as it can be observed in transiently transfected systems under stress as “maisonette squelching.” As follows, we assume that the slight increase in potency after co-transfection with **pRL-null[zfEF1a]** results from less competition between reporter and normalization vector (Fig. 5B). Further, we saw a strong increase in efficacy for **pGudluc7.5 + pRL-null[zfEF1aPro]** (Figs. 3 and 5A) but not for **pGL4.43 + pRL-null[zfEF1aPro]** (Fig. S5). Apparently, **pGL4.43** was not squelched in combination with the minP-bearing normalization vectors, whereas it was the case for **pGudluc7.5**. Hence, the utilization of a genomic promoter did not alter expression patterns significantly for the letter. However, **pGudluc7.5** profited substantially from co-transfection with **pRL-null[zfEF1aPro]**. We assume the increase in efficacy to be based on the higher amount of XREs (twenty within the promoter cassette of **pGudluc7.5**) compared to the synthetic promoter (three on **pGL4.43**; see also Table 1). Thus, once **pGudLuc7.5** is not squelched on the primary level, it can recruit a higher amount of AhR/ARNT and express the reporter gene more efficiently (see also paragraph above).

## Conclusion

Here, we report the development of an AhR-responsive transient reporter assay in the ZFL cell line by applying previously conceived technologies and strategies. Further, we report the vector geometry-induced squelching of the primary reporter signal by viral, constitutive promoters. Beyond, we designed a novel normalization vector, bearing an endogenous zebrafish-derived genomic promoter (zfEF1aPro), which rescues the squelching-delimited system, thus, giving new insights into the modulation of transient reporter systems under xenobiotic stress. Additionally, we confirmed in vivo results of the xenobiotic metabolism TP in zebrafish. Seemingly, zebrafish-derived systems are intrinsically low responders to AhR-mediated effects of dioxin-like compounds. Hence, their applicability is disputable in terms of predicting low-dose effects but valuable in mechanistic terms. Finally, we uncovered how the ubiquitously used ligand BNF promiscuously activates TPs of the xenobiotic metabolism and cellular stress response in an orchestral manner, leading to a concentration-related inhibition of the AhR/ARNT/XRE-TP and NMCRCs. We named such a multi-level inhibitory mechanism that might mask effects as “maisonette squelching.”

## Abbreviations

Most abbreviations are defined upon the first appearance, with some familiar exceptions.

A detailed list of abbreviations is given in the supplementary information file (SI)

## Supplementary Information

Below is the link to the electronic supplementary material.

Supplementary file1 (PDF 1.35 MB)

## Data Availability

All data generated or analyzed during this study are included in this published article (and its supplementary files). Raw data are available from the corresponding author upon request.
