# Supplementary material for: Development, scrutiny, and modulation of transient reporter gene assays of the xenobiotic metabolism pathway in zebrafish hepatocytes
Source: Cell Biol Toxicol. 2021 Oct 15;39(3):991–1013. doi: 10.1007/s10565-021-09659-0 (PMC10406726; doi:10.1007/s10565-021-09659-0)
Supplement: Supplementary file 1 — Supplementary file1 (PDF 1.35 MB) [file 10565_2021_9659_MOESM1_ESM.pdf]

Supplementary Information to

Development, scrutiny, and modulation of transient reporter gene assays of the xenobiotic metabolism pathway in zebrafish hepatocytes

Sebastian Lungu-Mitea*[a], Yuxin Han, and Johan Lundqvist[b]

Department of Biomedicine and Veterinary Public Health, Swedish University of Agricultural Sciences, Box 7028, SE-750 07 Uppsala, Sweden

* Corresponding author:
Sebastian Lungu-Mitea
E-mail: sebastian.lungu@slu.se
[a] https://orcid.org/0000-0001-8192-9134
[b] https://orcid.org/0000-0001-5693-9007

Contents

Supplementary Materials & Methods 2
Restriction enzyme cloning of pRL-null[zfEF1aPro] 2
Vector maps 3

Supplementary Results 5
Screening experiments after BNF exposure: additional data 5
Non-monotonous concentration response curves after BNF exposure: additional data 7
Sigmoidal concentration-response curves after TCDD exposure: additional data 8
ToxCast data 11
Induction of the oxidative stress response pathway (Nrf2) after BNF exposure 12

Abbreviations 15

References 16

Supplementary Materials & Methods

Restriction enzyme cloning of pRL-null[zfEF1aPro]

Primers for genomic amplification of zfEF1aPro cDNA, were synthesized as described in (Brautigam et al. 2013), supplementary information: zfEF1aPro_BglII_fwd (5'-CTGGAGGCCAGCTCAAACAT-3'); zfEF1aPro_XhoI_rev (5'-ATCAAGAAGAGTAGTACCGCTAGCATTAC-3').

The digestion mixture was prepared for both vectors in MilliQ-water (Merck, Darmstadt, Germany) containing acetylated-BSA, DNA buffer D and the BglII and XhoI digestion enzymes (all Promega, Madison, USA). The mixtures were incubated at 37°C for 4 hours, thermosensitive alkaline phosphatase (TSAP; Promega, Madison, USA) was added afterwards and incubated for additional 15 minutes at 37ºC to prevent self-ligation of the digested plasmids. The TSAP was further inactivated by incubation at 75ºC for 15 min.

Extraction and purification were conducted as follows: 1% agarose gel was prepared by mixing 1 g agarose (VWR, Pennsylvania, USA) with 100 ml 1X TAE buffer. The 1X TAE buffer was diluted from a 50X TAE buffer stock. An original stock solution was made by dissolving 242 g Tris base in MilliQ water, adding 57.1 ml glacial acetic acid, 100 ml of 500 mM EDTA solution, and adjusting the final volume to 1 L. 10000x GelRed Nucleic acid gel stain (VWR, Pennsylvania, USA) was added in a ratio of 1:10000 right before pouring the gel. 20 µl of the post-TSAP treatment digestion product were mixed with 6µl of DNA loading dye (Thermo Fisher, Vilnius, Lithuania) and transferred into the gel pockets. Electrophoresis ran for 30 min at 100 V, and the gel was visualized under UV light. The desired DNA fragments were cut out and transferred into a reaction tube. The DNA fragment was then purified using then Wizard® SV Gel and PCR Clean-up System (both Promega, Madison, USA) according to the manufacturer's protocol and subsequently stored in nuclease-free water at -20°C until further usage.

The desired fragments of **pRL-null** and "**ZF-L Exp**" were ligated using the LigaFastTM Rapid DNA Ligation System (Promega, Madison, USA) following the manufacturer's protocol.

The ligated product was transformed into DH5a competent bacteria (Sigma-Aldrich, Steinheim, Germany) and cultured in ampicillin containing LB agar plates. Single colonies were picked and further cultivated. The cultured plasmids were first test-extracted by using PureYield® Vector Mini-prep System (Promega, Madison, USA) according to the manufacturer’s protocol to determine the rate yield and to investigate the correct directional insertion of the zfEF1aPro sequence via diagnostic restriction digest and sequencing (TubeSeq service; Eurofins, Ebersberg, Germany). For final plasmid working stocks, midi-preps of the verified vectors were conducted and extracted using the NucleoBond® Xtra vector purification System (Macherey-Nagel, Düren, Germany) according to the manufacturer’s protocol. Fig S1 depicts a graphical illustration of the **pRL-null[zfEF1aPro]** construct.

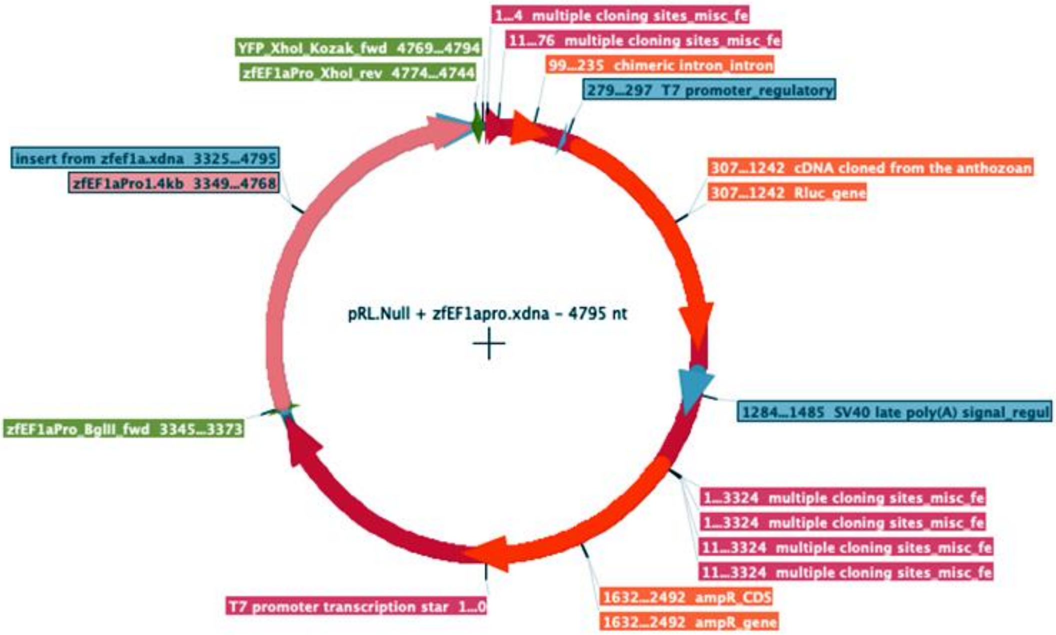

Fig. S1 Plasmid vector map of the pRL-null[zfEF1aPro] directional cloning product. Illustration created in SerialCloner 2.6.1.

Vector maps

Fig. S2 (below) Plasmid vector maps of applied reporter and normalization vectors used in co-transfection experiments, as summarized in tab. 1 (main manuscript). The sequence of the Firefly Luciferase reporter vector **pGudLuc7.5** is propietary concealed. Please consult the matrials and methods section of the main manuscript for major plasmid features (tab. 1). Illustrations were created in SerialCloner 2.6.1.

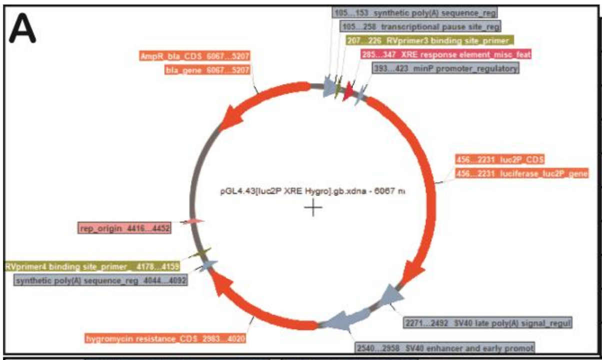

Plasmid vector	[promoter/enhancer]	Reporter
pGL4.43 (A)	[XRE]	Firefly
pGL4.70 (B)	[minP]	Renilla
pGL4.74 (C)	[TK]	Renilla
pGL4.73 (D)	[SV40]	Renilla
pGL4.75 (E)	[CMV]	Renilla
pRL-null (F)	[minP]	Renilla
pRL-TK (G)	[TK]	Renilla
pRL-SV40 (H)	[SV40]	Renilla
pRL-CMV (I)	[CMV]	Renilla

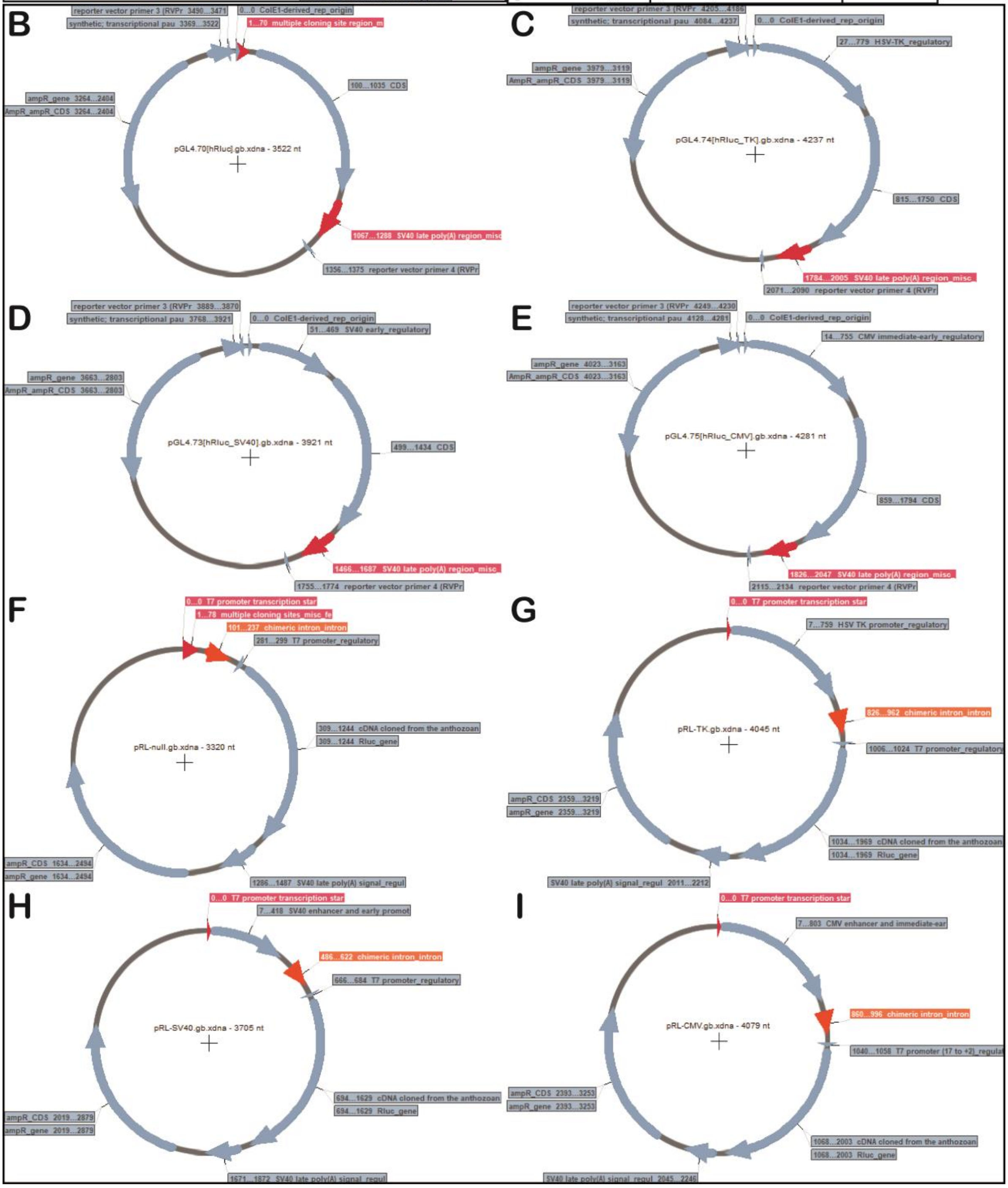

Supplementary Results

Screening experiments after BNF exposure: additional data

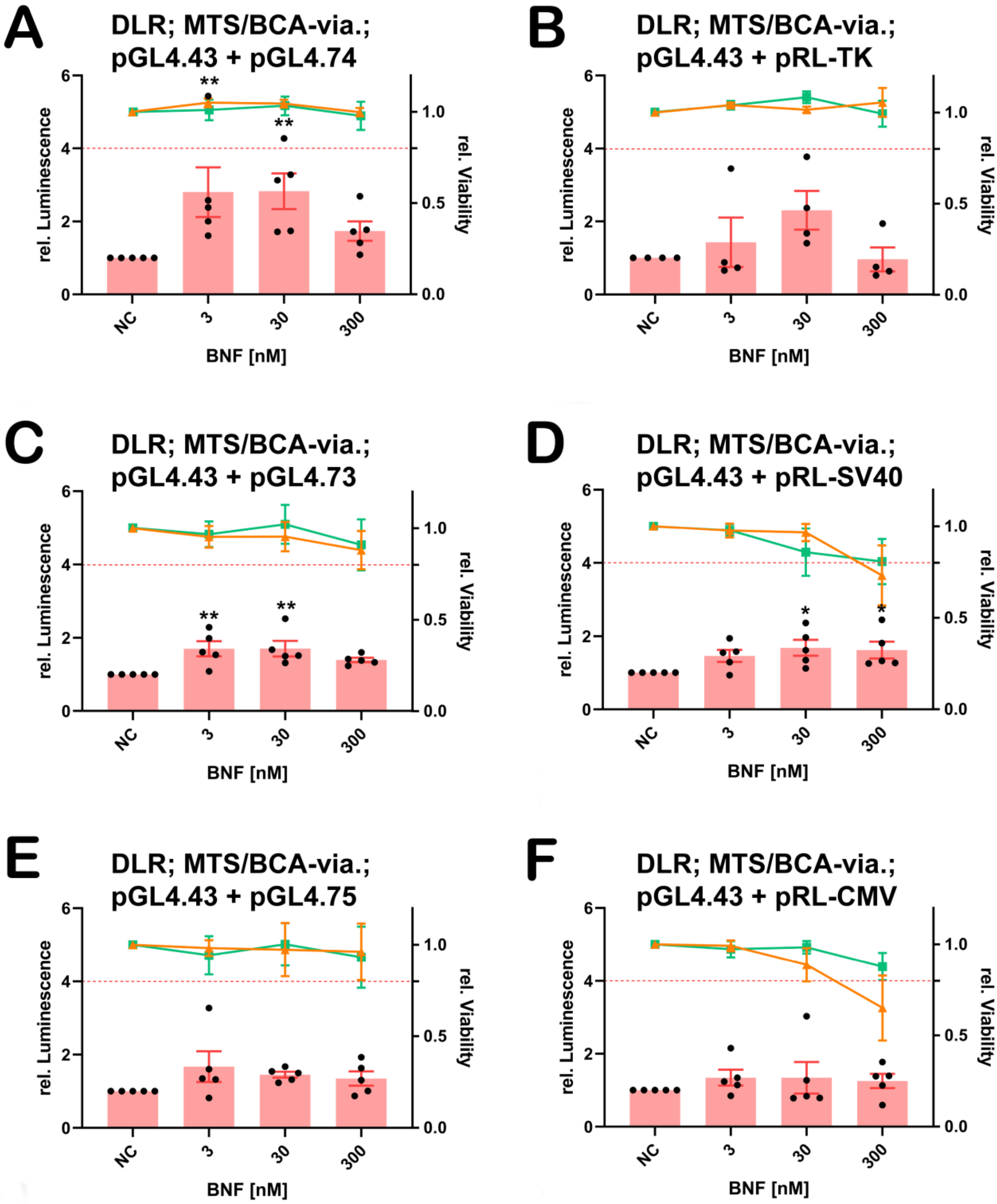

Fig. S3 Effects on luminescence measured in the ZFL cell line exposed to BNF. Luminescence corresponds to quantitative AhR transcription factor activation measured via DLR assay in cells co-transfected with the depicted combinations of reporter and normalization vectors (A-F; all viral promoters). Mean normalized luminescence induction is illustrated as red bars, black dots represent means of single experiments, red whiskers represent the

SEM (experimental units n = 4-5; observational units N = 12-15). Cellular viability corresponds to apical endpoints measured via the MTS/BCA-multiplex assay. Each point (MTS orange, BCA green) represents the mean, including SEM (experimental units n = 4-5; observational units N = 12-15). A threshold value of 0.8 was considered biologically significant (dotted red line). Asterisks indicate significance tested in a one-way ANOVA with Dunnett's post hoc test (*P < 0.05, **P < 0.01, ***P < 0.001).

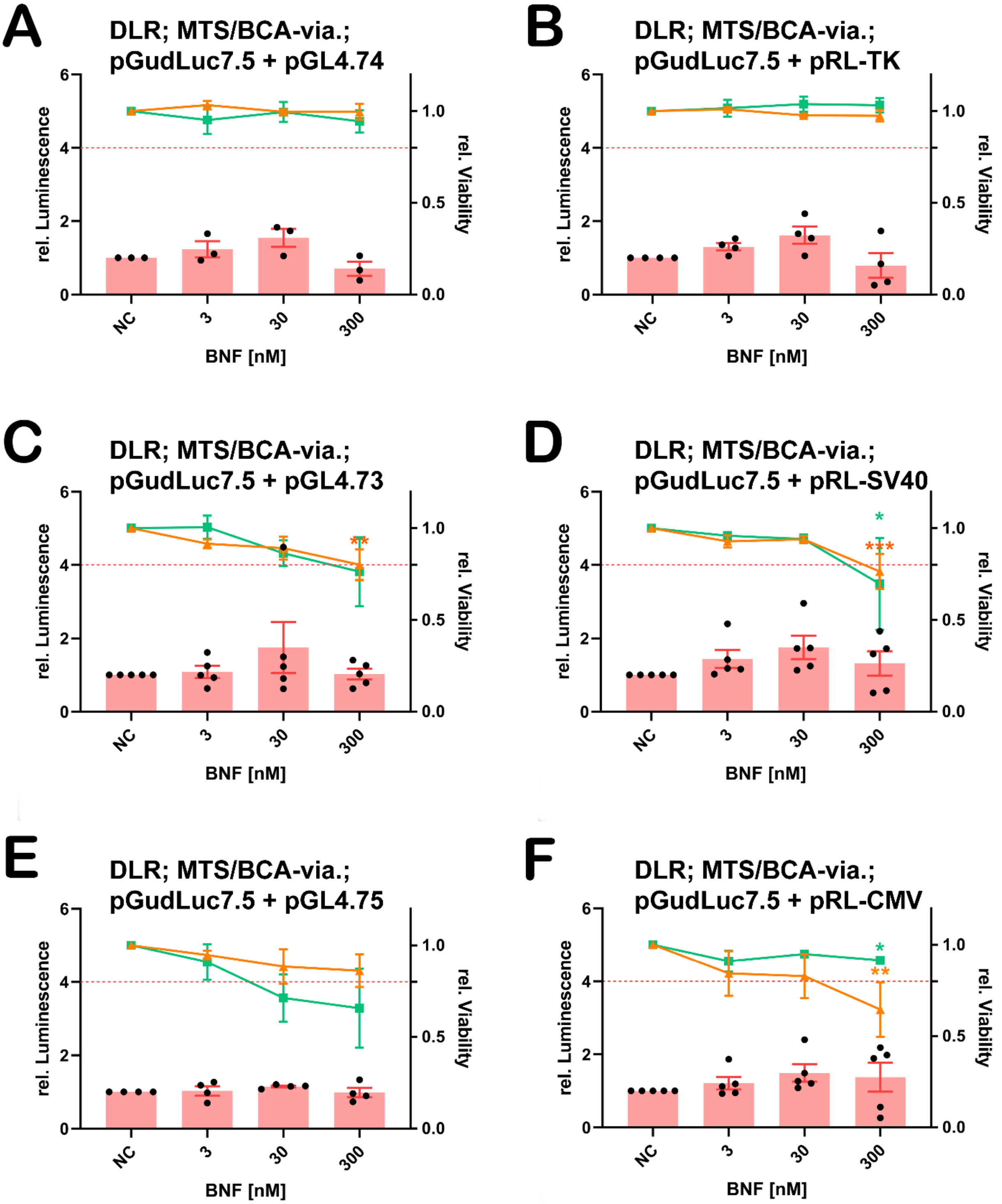

Fig. S4 Effects on luminescence measured in the ZFL cell line exposed to BNF. Luminescence corresponds to quantitative AhR transcription factor activation measured via DLR assay in cells co-transfected with the depicted combinations of reporter and normalization vectors (A-F; all viral promoters). Mean normalized luminescence induction is illustrated as red bars, black dots represent means of single experiments, red whiskers represent the SEM (experimental units n = 3-5; observational units N = 9-15). Cellular viability corresponds to apical endpoints

measured via the MTS/BCA-multiplex assay. Each point (MTS orange, BCA green) represents the mean, including SEM (experimental units n = 3-5; observational units N = 9-15). A threshold value of 0.8 was considered biologically significant (dotted red line). Asterisks indicate significance tested in a one-way ANOVA with Dunnett's post hoc test (*P < 0.05, **P < 0.01, ***P < 0.001).

Non-monotonous concentration response curves after BNF exposure: additional data

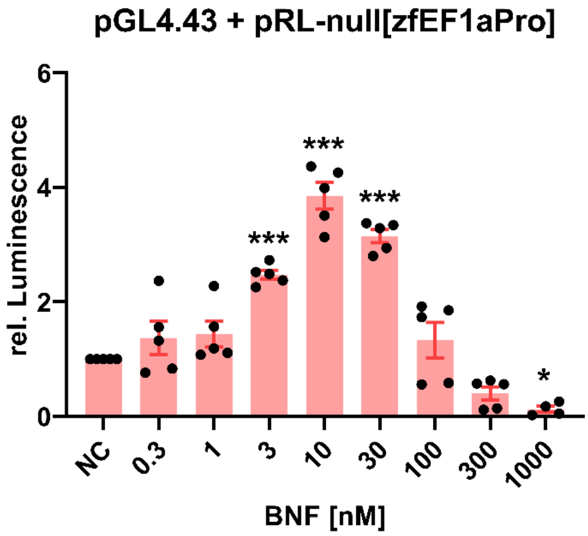

Fig. S5 Effects on luminescence measured in the ZFL cell line exposed to BNF. Luminescence corresponds to quantitative AhR transcription factor activation measured via DLR assay in cells co-transfected with the **pGL4.43 + pRL-null[zfEF1aPro]** co-transfection setup. Mean normalized luminescence induction is illustrated as red bars, black dots represent means of single experiments, red whiskers represent the SEM (experimental units n = 5; observational units N = 15).

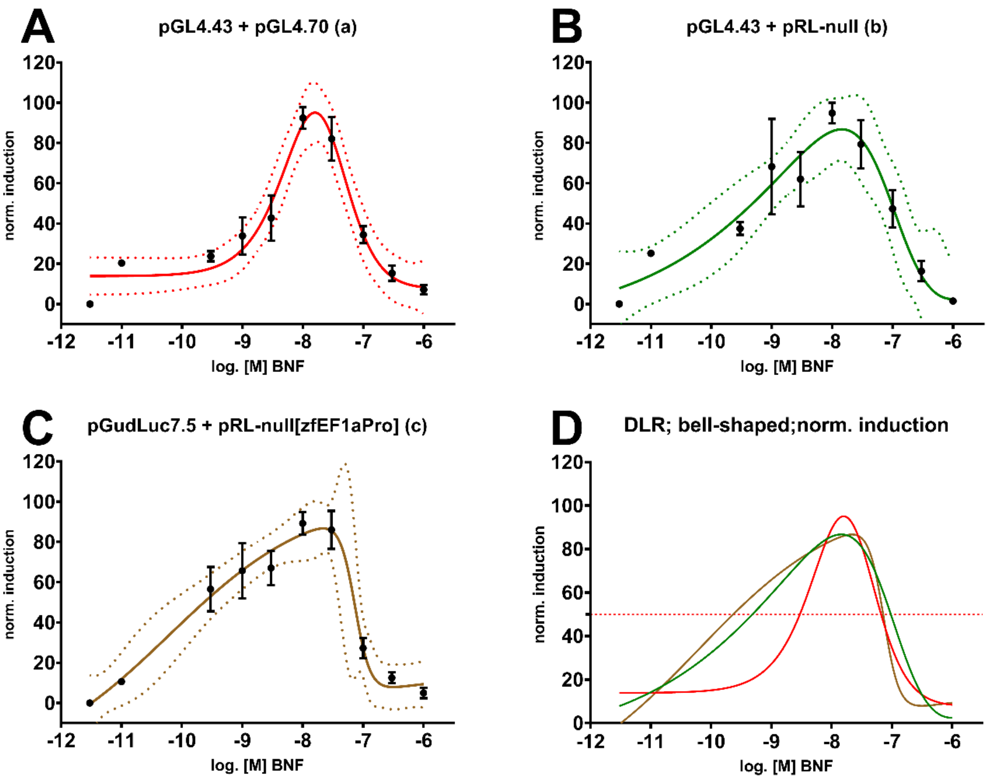

Fig. S6 Bell-shaped concentration-response curves of depicted co-transfection setups after BNF exposure. Results of the DLR assays were fitted as normalized induction values to a bell-shaped nonlinear regression model. EC_{50}/IC_{50} values are summarized in tab. 2 (main article). Depicted data points for every concentration are the mean of means (experimental unit n = 3-5; observational unit N = 9-15), including SEM. Continuous lines depict the computed regression lines, and dotted lines represent the 95% CI.

Sigmoidal concentration-response curves after TCDD exposure: additional data

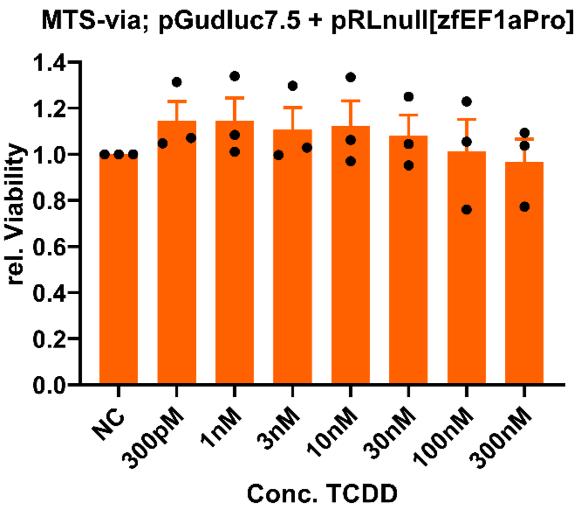

Fig. S7 Cellular viability in ZFL cell after co-transfection with the **pGudLuc7.5** and **pRL-null[zfEF1aPro]** vector combination and exposure to depicted concentrations of TCDD. Cellular viability corresponds to NADPH metabolism turnover measured via the MTS assay. Each bar represents the mean, including SEM (experimental units n=3; observational units N=9). A one-way ANOVA with Dunnett's post hoc test was conducted, but no statistical significance was computed.

Tab. S1 LOEC and $EC_{20/50}$ values derived from TCDD exposure (adj. R^2 and NRSME values are given as goodness-of-fit parameters of the nonlinear regression). See respective illustrations in fig. S8.

**Transfection setup** (according to fig. 4)	**LOEC** (adj. P-value)	**EC_{20}** (normalized; adj. R^2; NRSME)	**EC_{50}** (normalized; adj. R^2; NRSME)	**EC_{50}** (relative; non-normalized; adj R^2; NRSME)	**N** (exp.)
**(a) pGL4.43 + pGL4.70**	100 pM (0.0406)	166 pM (0.88; 0.19)	612 pM (0.88; 0.19)	595 pM (0.57; 0.29)	8
**(b) pGL4.43 + pRL-null**	300 pM (0.008)	297 pM (0.85; 0.28)	2.70 nM (0.85; 0.28)	1.31 nM (0.47; 0.11)	6
**(c) pGudLuc7.5 + pRL-null[zfEF1aPro]**	100 pM (0.0003)	287 pM (0.92; 0.16)	1.15 nM (0.92; 0.16)	1.13 nM (0.62; 0.11)	8
**(d) pGL4.43 + pRL-null[zfEF1aPro]**	100 pM (0.0083)	282 pM (0.89; 0.16)	1.45 nM (0.89; 0.16)	877 pM (0.61; 0.32)	9
**(e) pGudLuc7.5 + pRL-null**	1 nM (0.0004)	245 pM (0.86; 0.39)	661 pM (0.86; 0.40)	685 pM (0.75; 0.31)	4
**(f) pGudLuc7.5 + pGL4.70**	1 nM (0.0002)	204 pM (0.78; 0.68)	1.26 nM (0,81; 0.68)	1.07 nM (0.68; 0.05)	3

Tab. S2 Comparison of mean co-transfection setup effects between combinations depicted in fig. S8 and tab. S1. Statistical significance in mean setup effect was assessed via two-way ANOVA, followed by Tukey's post hoc test (*P < 0.05, **P < 0.01, ***P < 0.001). Non-significant combinations are not depicted.

**Comparison**	**Adjusted P-value**	**Significance**
**a vs. b** (rel.)	0.0015	**
**a vs. c** (rel.)	<0.0001	***
**a vs. f** (rel.)	0.0234	*
**b vs. c** (rel.)	<0.0001	***
**b vs. e** (rel.)	<0.0001	***
**b vs. f** (rel.)	<0.0001	***
**c vs. d** (rel.)	<0.0001	***
**c vs. e** (rel.)	<0.0001	***
**c vs. f** (rel.)	<0.0001	***
**d vs. f** (rel.)	0.0006	***
**a vs. b** (norm.)	0.0025	**
**a vs. d** (norm.)	0.0031	**

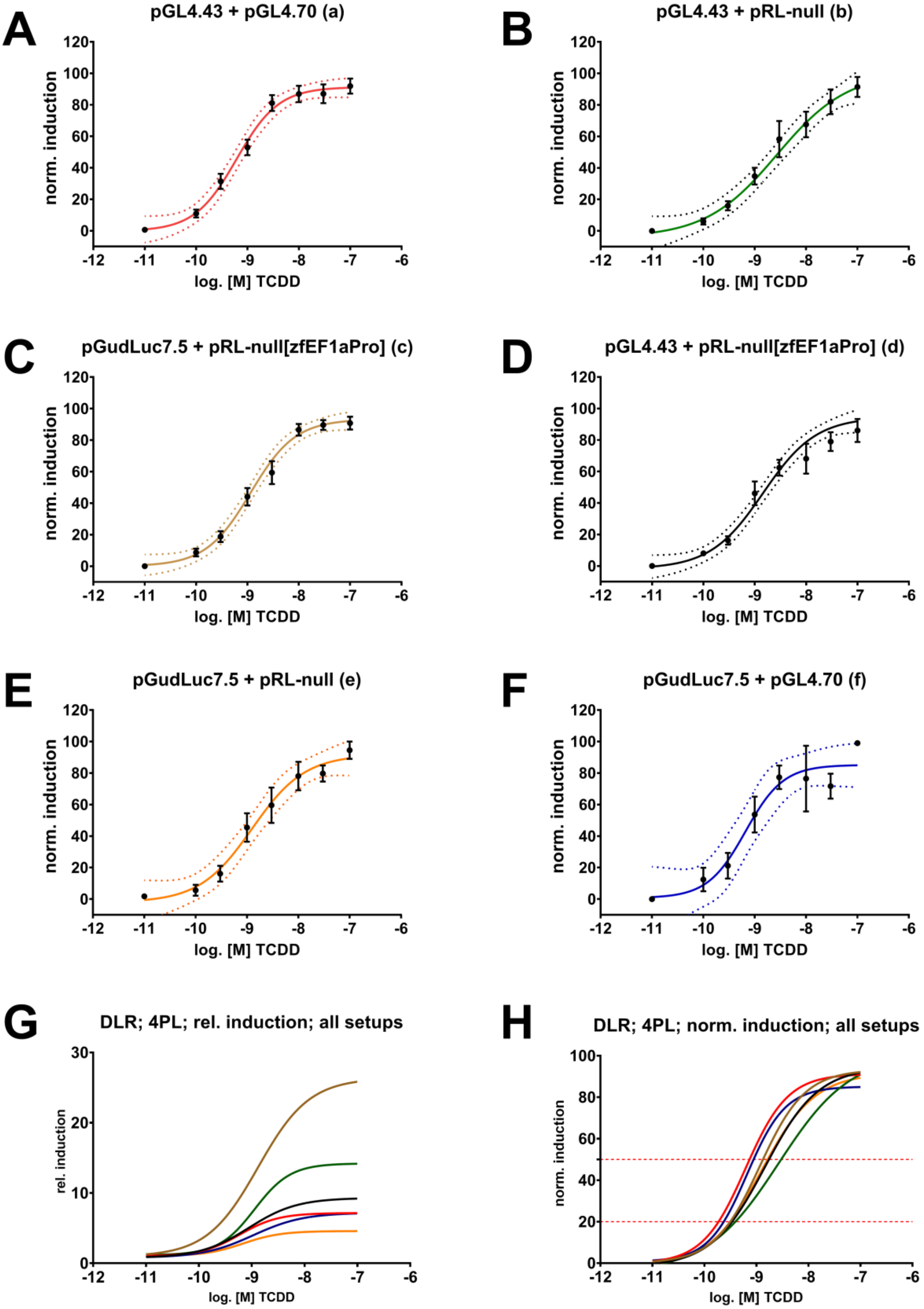

Fig. S8 Concentration-response curves of depicted co-transfection setups after TCDD exposure. Results of the DLR assays were fitted as normalized induction values to a four-parameter log-logistic nonlinear regression model. $EC_{20/50}$ values are summarized in tab. S1. Depicted data points for every concentration are the mean of means (experimental unit n = 3-9; observational unit N = 9-27), including SEM. Continuous lines define the computed regression lines, and dotted lines represent the 95% CI.

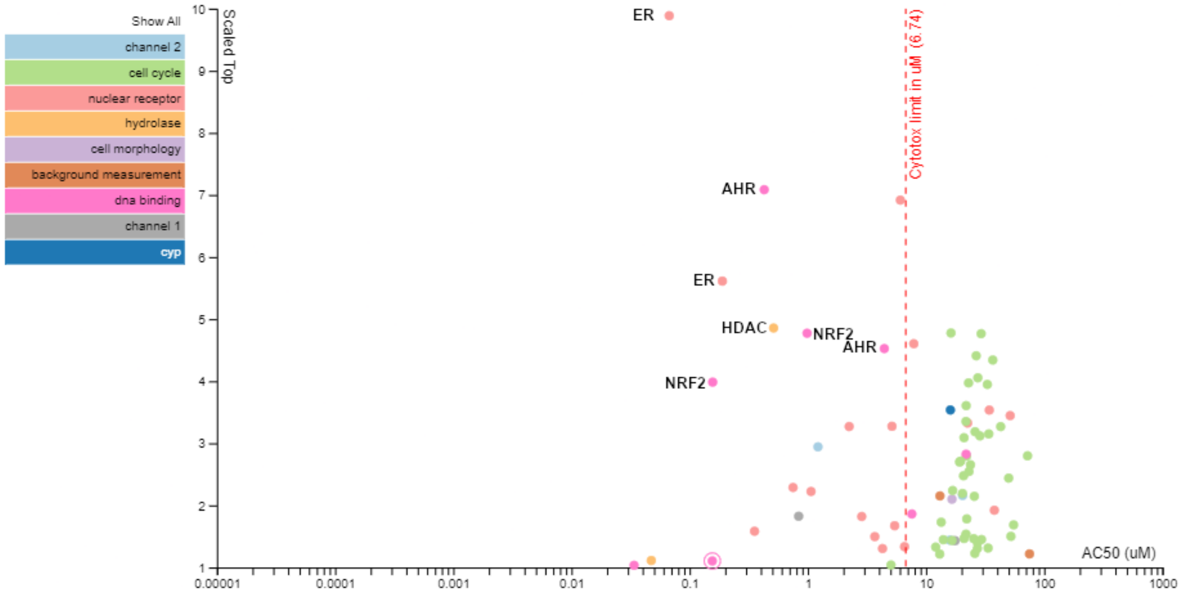

Fig. S9 Bioactivity data of BNF in the ToxCast database. Illustration generated in the CompTox chemical dashboard (https://comptox.epa.gov/dashboard) and modified. See tab. S3 for the specifically induced bioassays.

Tab. S3 Excerpt of ToxCast database data acquired in the Comptox dashboard. Positive bioassay hits after BNF exposure. Bioassay hits were cut off at the cytotoxicity limit and sorted by effect concentrations (activity concentration 50% – AC_{50}).

Bioassay	Symbol	Gene	AC_{50} [µM]	Target
TOX21_SSH_3T3_GLI3_Agonist	–	–	3.39E-02	DNA binding
TOX21_ELG1_LUC_Agonist	ATAD5	ATPase family, AAA domain containing 5	4.74E-02	hydrolase
TOX21_ERR_Agonist	ESRRA	estrogen-related receptor alpha	6.72E-02	nuclear receptor
ATG_AP_1_CIS_up	FOS	FBJ murine osteosarcoma viral oncogene homolog	0.156	DNA binding
ATG_NRF2_ARE_CIS_up	NFE2L2	nuclear factor, erythroid 2-like 2	0.157	DNA binding
TOX21_PGC_ERR_Agonist	ESRRA	estrogen-related receptor alpha	0.189	nuclear receptor
TOX21_RAR_LUC_Agonist	RARA	retinoic acid receptor, alpha	0.354	nuclear receptor
ATG_Ahr_CIS_up	AHR	aryl hydrocarbon receptor	0.428	DNA binding
TOX21_HDAC_Inhibition	–	–	0.513	hydrolase
ATG_PPARg_TRANS_up	PPARG	peroxisome proliferator-activated receptor gamma	0.75	nuclear receptor
TOX21_ARE_BLA_Agonist_ch1	–	–	0.837	channel 1

TOX21_ARE_BLA_agonist_ratio	NFE2L2	nuclear factor, erythroid 2-like 2	0.985	DNA binding
ATG_ERE_CIS_up	ESR1	estrogen receptor 1	1.06	nuclear receptor
TOX21_ARE_BLA_Agonist_ch2	–	–	1.22	channel 2
ATG_PXRE_CIS_up	NR1I2	nuclear receptor subfamily 1, group I, member 2	2.24	nuclear receptor
ATG_ERa_TRANS_up	ESR1	estrogen receptor 1	2.86	nuclear receptor
TOX21_AR_LUC_MDAKB2_Agonist_3uM_Nilutamide	AR	androgen receptor	3.69	nuclear receptor
ATG_VDRE_CIS_up	VDR	vitamin D (1,25-dihydroxyvitamin D3) receptor	4.28	nuclear receptor
TOX21_AhR_LUC_Agonist	AHR	aryl hydrocarbon receptor	4.44	DNA binding
TOX21_RT_HEK293_FLO_40hr_viability	–	–	5.05	cell cycle
TOX21_ERa_LUC_VM7_Agonist	ESR1	estrogen receptor 1	5.15	nuclear receptor
TOX21_AR_LUC_MDAKB2_Agonist	AR	androgen receptor	5.43	nuclear receptor
TOX21_CAR_Agonist	NR1I3	nuclear receptor subfamily 1, group I, member 3	6.07	nuclear receptor
ATG_PXR_TRANS_up	NR1I2	nuclear receptor subfamily 1, group I, member 2	6.58	nuclear receptor

Induction of the oxidative stress response pathway (Nrf2) after BNF exposure

To undermine the "*maisonette*" squelching hypothesis, Comptox data regarding the induction of cellular stress response pathways were backed up by empirical data in ZFL cell lines. Therefore, ZFL cells were transiently co-transfected with the Nrf2-responsive reporter vector **pGL4.37** and the normalization vector **pRL-CMV**, as described in (Lungu-Mitea and Lundqvist 2020). Experimental design, data handling, and statistical evaluation were identical to procedures described in the main manuscript. The transcription factor Nrf2 is a central regulator of the oxidative stress response pathways (Nrf2/Keap1/ARE) (Itoh et al. 2004) and a keystone in the regulation of oxidative stress detoxification, metabolism, and induction of related phase-II enzymes (e.g. GST, NQO1, HO-1).

The results are given in figs. S10 and S11. LOEC Nrf2 induction was recorded at 30 nM BNF (fig. S10). The maximal induction of 3.4 fold was measured at a concentration of 100 nM BNF. Statistically significant cytotoxicity was recorded from 300 nM BNF onwards, coinciding with a down-regulation of the Nrf2-related luciferase signal. Crystalized aggregates were encountered above 10 µM BNF upon optical inspection under the microscope (not illustrated), leading to artefact effects in the luciferase measurement. Therefore, specific points were excluded from the analysis and deemphasized in fig. S10. Possibly, compound aggregation led to a flattening of the cytotoxicity curve in fig. S10. Compound aggregation at higher molar concentrations has been discussed in (Owen et al. 2012, 2014). Concentrations up to cytotoxicity were used to derive a sigmoidal concentration-response relationship (fig. S11). The computed parameters are depicted in tab. S4.

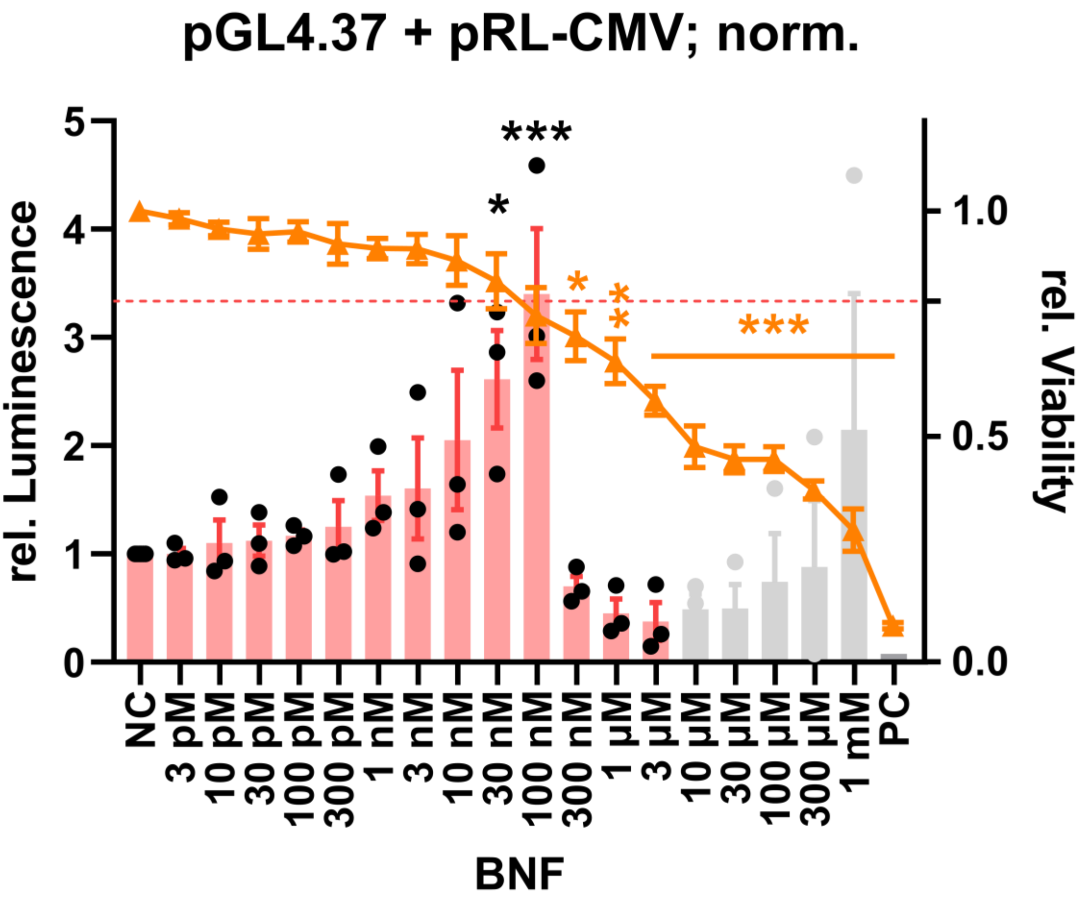

Fig. S10 Effects on luminescence measured in the ZFL cell line exposed to BNF. Luminescence corresponds to quantitative Nrf2 transcription factor activation measured via the DLR assay in cells co-transfected with the depicted combination of reporter and normalization vectors. Mean normalized luminescence induction is illustrated as red bars, black dots represent means of single experiments, red whiskers represent the SEM (experimental units n = 3; observational units N = 9). Cellular viability corresponds to apical endpoints measured via the MTS assay. 10% (v/v) DMSO was used as a positive control (PC). Each orange triangle represents the mean, including SEM (experimental units n = 3; observational units N = 9). A threshold value of 0.8 was considered biologically significant (dotted red line). Asterisks indicate significance tested in a one-way ANOVA with Dunnett's post hoc test (*P < 0.05, **P < 0.01, ***P < 0.001).

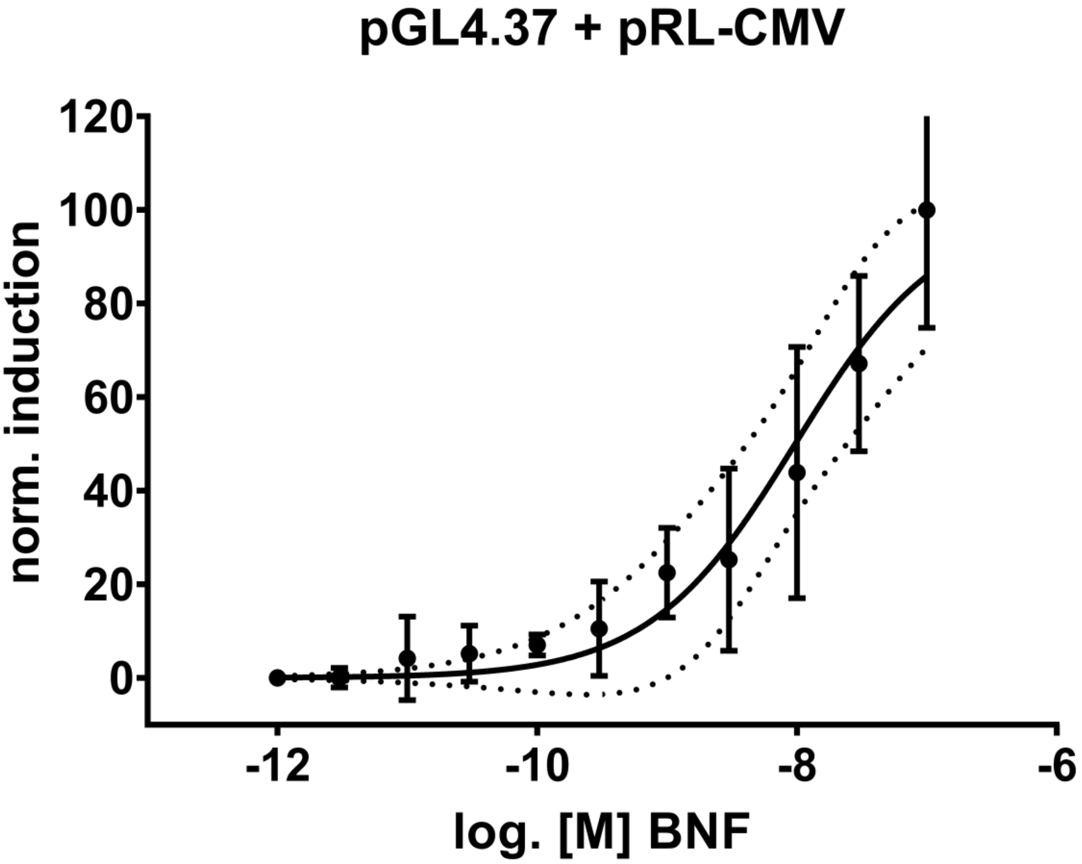

Fig. S11 Concentration-response curves of the depicted co-transfection setup after BNF exposure. Results of the DLR assay were fitted as normalized induction values to a four-parameter log-logistic nonlinear regression model. Derived parameters are summarized in tab. S4. Depicted data points for every concentration are the mean of means (experimental unit n = 3; observational unit N = 9), including SEM. Continuous lines define the computed regression lines, and dotted lines represent the 95% CI.

Tab. S4 Computed parameters of the 4PL nonlinear regression model. Transiently transfected cells (see fig. S11) were exposed to increasing concentrations of BNF.

**EC_{50} (CI)**	**Slope (CI)**	**Adjusted R^2**	**NRMSE**
9.68 nM (4.2-21.6 nM)	0.77 (0.43-1.62)	0.65	0.61

Abbreviations

Abbreviation	Explanation
3Rs	Refine, reduce, replace (animal tests)
AhR	aryl hydrocarbon receptor
AOP	adverse outcome pathway
AR	Androgen receptor
ARE	Anti-oxygen response element
ARNT	aryl hydrocarbon receptor nuclear translocator
BNF	β-naphthoflavone
CRC	Concentration-response curve
DLC	Dioxin-like compound
DLR	Dual reporter gene assay
ECx	Concentration causing x% of effect
ER	Estrogen receptor
FET	fish embryo test
Fluc	Firefly luciferase
GR	glucocorticoid receptor
GST	Glutathione S-transferase
HIF-1	hypoxia inducible factor 1
HO-1	Heme oxygenase 1
ICx	Concentration causing x% of inhibition
IVIVE	in vitro to in vivo extrapolation
Keap1	Kelch-like ECH-associated protein 1
LOEC	Lowest observed effect concentration
MAPK	mitogen-activated protein kinases pathway
MCS	Multiple cloning site
MIE	molecular initiating events
MOA	mechanism of action
MTF	Metallothionein transcription factor
NAM	new approach method
NfkB	nuclear factor-kappa B
NMCRCs	Non-monotonous concentration-response curves
NOEC	No observed effect concentration
NQO1	NAD(P)H dehydrogenase [quinone] 1
Nrf2	Nuclear factor erythroid 2-related factor 2
P53	Cellular tumor antigen p53
PAH	polycyclic aromatic hydrocarbon
PPAR	peroxisome proliferator-activated receptors

PXR	pregnane X receptor
RAR	retinoic acid receptor
Rluc	Renilla luciferase
TCDD	2,3,7,8-tetrachlorodibenzo-p-dioxin
Tox21	toxicology in the 21st century framework
TP	toxicity pathway
VDR	vitamin D receptor
WFD	Water Framework Directive
XRE	xenobiotic response element (aka. dioxin response element DRE)
ZFL	Zebrafish liver cell line

References

Brautigam L, Jensen LDE, Poschmann G, et al (2013) Glutaredoxin regulates vascular development by reversible glutathionylation of sirtuin 1. Proc Natl Acad Sci 110:20057–20062. https://doi.org/10.1073/pnas.1313753110

Itoh K, Tong KI, Yamamoto M (2004) Molecular mechanism activating Nrf2-Keap1 pathway in regulation of adaptive response to electrophiles. Free Radic Biol Med 36:1208–13. https://doi.org/10.1016/j.freeradbiomed.2004.02.075

Lungu-Mitea S, Lundqvist J (2020) Potentials and pitfalls of transient in vitro reporter bioassays: interference by vector geometry and cytotoxicity in recombinant zebrafish cell lines. Arch Toxicol. https://doi.org/10.1007/s00204-020-02783-6

Owen SC, Doak AK, Ganesh AN, et al (2014) Colloidal Drug Formulations Can Explain “Bell-Shaped” Concentration–Response Curves. ACS Chem Biol 9:777–784. https://doi.org/10.1021/cb4007584

Owen SC, Doak AK, Wassam P, et al (2012) Colloidal aggregation affects the efficacy of anticancer drugs in cell culture. ACS Chem Biol 7:1429–1435. https://doi.org/10.1021/cb300189b
